# Identification and characterization of mono- and bifunctional galactan synthases in the pediatric pathogen *Kingella kingae*

**DOI:** 10.1016/j.jbc.2025.110345

**Published:** 2025-06-06

**Authors:** Eric A. Porsch, Mikel Jason Allas, Nina R. Montoya, Vanessa L. Muñoz, Li Tan, Artur Muszyński, Parastoo Azadi, Stephen N. Hyland, Catherine L. Grimes, Tzu-Ting Kao, Todd L. Lowary, Joseph W. St. Geme

**Affiliations:** 1 Department of Pediatrics, Children’s Hospital of Philadelphia, Philadelphia, Pennsylvania, USA; 2 Department of Chemistry, University of Alberta, Edmonton, Alberta, Canada; 3 Institute of Biological Chemistry, Academia Sinica, Nangang, Taipei, Taiwan; 4 Perelman School of Medicine, University of Pennsylvania, Philadelphia, Pennsylvania, USA; 5 Complex Carbohydrate Research Center, University of Georgia, Athens, Georgia, USA; 6 Department of Chemistry and Biochemistry, University of Delaware, Newark, Delaware, USA; 7 Department of Biological Sciences, University of Delaware, Newark, Delaware, USA; 8 Institute of Biochemical Sciences, National Taiwan University, Taipei, Taiwan

**Keywords:** Gram-negative bacteria, glycosyltransferase, galactan, lipopolysaccharide, galactose, galactofuranose, synthase

## Abstract

The emerging pediatric pathogen *Kingella kingae* elaborates a lipopolysaccharide (LPS) that is extended with a galactofuranose homopolymer called galactan, which is a key virulence determinant that contributes to resistance to complement-mediated and neutrophil-mediated killing. Previous work has demonstrated that the *pamABCDE* locus is required for galactan synthesis. In this study, mutational studies suggested that the *pamC* gene product is a UDP-galactofuranose (Gal*f*) transferase and is the galactan synthase. Analysis of genome sequence data revealed two distinct *pamC* alleles designated *pamC1* and *pamC2*, which correlate with the two galactan structures in *K. kingae*. Examination of isogenic mutants expressing either *pamC1* or *pamC2* demonstrated that the *pamC* alleles are the determinants of galactan structure. Experiments with recombinant PamC1 and PamC2 *in vitro* established that these proteins are galactan synthases capable of extending synthetic Gal*f* disaccharide acceptors in the presence of UDP-Gal*f*. Homology analysis identified critical amino acids that are essential for PamC1 and PamC2 enzymatic activity both *in vitro* and in *K. kingae*. Structural analysis of the *in vitro*-modified synthetic acceptors implicated PamC1 as a monofunctional enzyme capable of generating a **β**-(1 → 5) Gal*f* linkage and PamC2 as a bifunctional enzyme capable of generating **β**-(1 → 3) and **β**-(1 → 6) Gal*f* linkages. This study advances our understanding of the GT2 family of UDP-galactofuranosyltransferases.

The cell surfaces of Gram-negative bacteria are typically decorated with a variety of carbohydrate structures of varying complexity, including capsular polysaccharides, exopolysaccharides, and lipopolysaccharides (LPSs). These structures exhibit diverse functions, including preventing desiccation, promoting biofilm formation, and mediating serum resistance, among others. The pediatric pathogen *Kingella kingae* has been demonstrated to produce an extracellular galactofuranose (Gal*f*) homopolymer called galactan that is composed of a linear chain of a β-(1 → 5) linked Gal*f* residues (1) or a disaccharide homopolymer of alternating β-(1 → 3) and β-(1 → 6) Gal*f* (2) (Fig. 1*A*), depending on the strain. Galactan was originally described as an exopolysaccharide with anti-biofilm properties (2). Further functional analysis revealed that galactan possesses important virulence properties, mediating resistance to opsonin deposition (3), complement-mediated killing (3, 4), and neutrophil killing (4). Galactan was also found to be required for *K. kingae* virulence in a juvenile rat model of invasive disease (3). Recent genetic, structural, and biochemical analyses revealed that galactan is not freely secreted but rather is linked to LPS, which also contains an uncharacterized O-antigen-like polymer (5). The precise glycosidic linkage between the galactan and LPS has not been determined.

**Figure 1:**
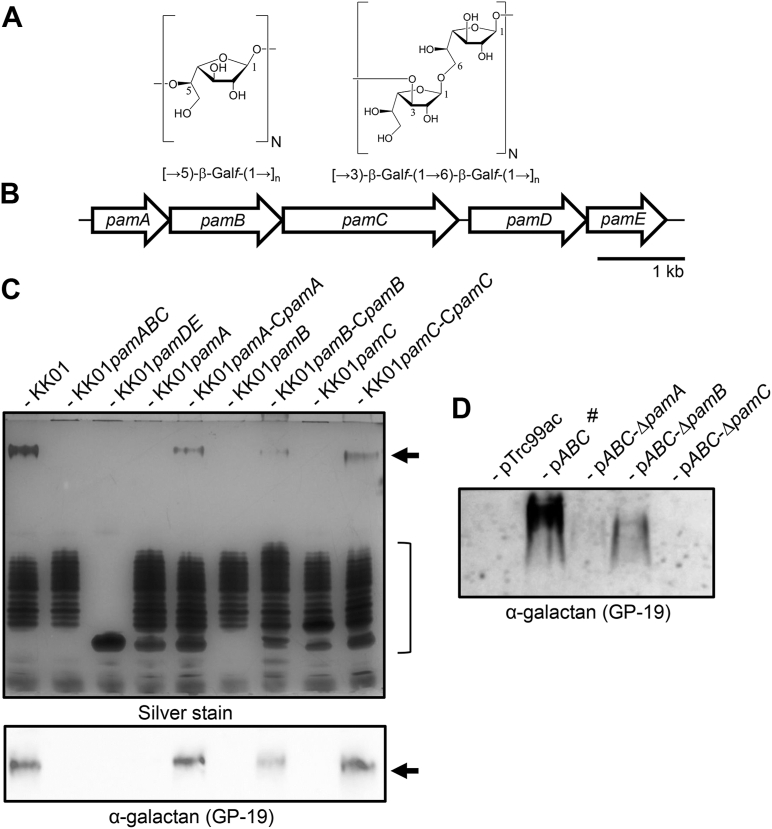
**Essential role of *pamA*, *pamB,* and *pamC* for galactan production in *K. kingae* and of *pamC* for galactan production in *E. coli*.***A*, The chemical structures of the [→5)-β-Gal*f*-(1→] polymer and the [→3)-β-Gal*f*-(1 → 6)-β-Gal*f*-(1→] polymer are shown. *B,* diagram of the *pamABCDE* locus. The *pamA* gene is predicted to encode a GT111 UDP-galactofuranose transferase, *pamB* is predicted to encode a UDP-galactopyranose mutase, *pamC* is predicted to encode a GT2 family UDP-galactofuranose transferase, *pamD* is predicted to encode a GT-B family glycosyltransferase, and *pamE* is predicted to encode a GT25 family glycosyltransferase. *C,top panel*, the migration profiles of LPS from strain KK01 and mutants KK01*pamABC*, KK01*pamDE*, KK01*pamA,* KK01*pamB*, and KK01*pamC* along with complemented strains KK01*pamA-CpamA*, KK01*pamB-CpamB*, and KK01*pamC-CpamC* following DOC-PAGE separation and silver staining are shown. The *black arrow* indicates the high-molecular-weight (HMW) LPS species containing galactan, and the bracket indicates the low-molecular-weight (LMW) LPS species lacking the galactan. *C,bottom panel*, the same LPS samples as in the *top panel* were separated using DOC-PAGE, transferred to nitrocellulose, and probed with GP-19, an anti-galactan guinea pig antiserum generated against a glycoconjugate containing the galactan from strain KK01 (5). The *black arrow* indicates reactivity with the HMW LPS species. *D*, Whole cell lysates of *E. coli* JM109 containing plasmids Trc99a (vector only control), pTrc-*pamABC* (pABC), pTrc-*pamABCΔpamA* (pABC-*ΔpamA*), pTrc-*pamABCΔpamB* (pABC-*ΔpamB*), and pTrc-*pamABCΔpamC* (pABC-*ΔpamC*) were treated with Proteinase K, separated with DOC-PAGE, transferred to nitrocellulose, and probed with GP-19. The GP-19 reactive material was detected at the *top* of the resolving portion of the gel, indicating a high molecular weight. #, only 25% of the relative sample volume for pABC was loaded compared to the other samples to account for the large amount of galactan produced in this strain.

LPS is ubiquitous in Gram-negative bacteria and is the main constituent of the outer leaflet of the outer membrane. The LPS molecule is a tripartite structure that contains lipid A, which anchors the molecule in the outer membrane, a core oligosaccharide region of non-repeating glycosyl residues, which is linked to lipid A, and a polysaccharide made up of repeating sugar units called the O-antigen, which is linked to the core region and extends into the extracellular environment (reviewed in (6)). Some bacteria produce a lipooligosaccharide (LOS), which contains lipid A and a core oligosaccharide that is commonly extended with a limited number of non-repeating sugars to form an outer core region but lacks a classical O-antigen (7). While there are structurally conserved elements in lipid A and the core oligosaccharide, there is considerable inter- and intra-species heterogeneity in O-antigen structure in LPS-producing bacteria and in the outer core region in LOS-producing bacteria. LPS and LOS have been extensively studied in a variety of pathogenic bacteria and are associated with numerous virulence properties.

In *K. kingae*, the genetic locus implicated in galactan production contains five genes, called *pamA*, *pamB, pamC, pamD,* and *pamE* (1, 2, 5) (Fig. 1*B*). Four of these genes encode predicted glycosyltransferases (PamA, PamC, PamE, and PamD), and the fifth encodes a predicted UDP-galactopyranose mutase (PamB) (1, 2, 5). Analysis of LPS from a *pamABC* mutant revealed loss of galactan but retention of the O-antigen-like structure (5). In contrast, LPS from a *pamDE* mutant lacked both galactan and the O-antigen-like structure. Expression of just *pamABC* in *Escherichia coli* resulted in the production of intracellular galactan (2, 5). Taken together, these results suggest that PamA, PamB, and PamC are sufficient for the synthesis of galactan, assuming the presence of the necessary substrates, and that PamD and PamE are necessary for O-antigen and galactan modification of LPS in *K. kingae*.

In this work, we set out to define the galactan synthesis pathway in *K. kingae* and to biochemically characterize the galactan synthase. Our results established that PamC is the galactan synthase. Further analysis revealed PamC variants designated PamC1 and PamC2 that correlate with galactan structure, with PamC1 generating the [→5)-β-Gal*f*-(1→]_n_ polymer (type 1 galactan) and PamC2 generating the [→3)-β-Gal*f*-(1 → 6)-β-Gal*f*-(1→]_n_ polymer (type 2 galactan).

## Results

### PamA and PamC are predicted UDP-galactofuranosyltransferases, but only PamC is essential for galactan synthesis when PamABC are expressed in *E. coli*

Based on the previous findings that *pamABC* are sufficient for galactan synthesis when they are co-expressed in *E*. *coli* (2, 5), and that deletion of *pamABC* in *K. kingae* abrogates galactan production (5), we hypothesized that one or more of the products of the *pamA, pamB,* and *pamC* genes is the enzyme responsible for the synthesis of the galactan polymer. To understand the putative biochemical roles of PamA, PamB, and PamC in galactan biosynthesis, we performed bioinformatic analyses based on primary amino acid sequence homology using BLASTp (8) and structural predictions using AlphaFold 3 (9). Of the *pamABC* gene products, PamB has the strongest similarity scores and is predicted to be a UDP-galactopyranose (Gal*p*) mutase, an enzyme that catalyzes the conversion between UDP-Gal*p* and UDP-Gal*f* (10). An overlay of the AlphaFold 3 PamB structural prediction with the crystal structure of the *E. coli* UDP-Gal*p* mutase is shown in Fig. S1*A*. Using an *in vitro* assay with purified recombinant PamB, we found that PamB converts UDP-Gal*f* to UDP-Gal*p,* confirming that PamB is a UDP-Gal*p* mutase (Fig. S2). Bioinformatic analysis of PamA revealed a domain of unknown function called DUF4422. A recent study by Clarke *et al.* (11) demonstrated that this domain is present in the N-terminal region of the *Klebsiella pneumoniae* serotype O2a WbbM O-antigen glycosyltransferase, which has UDP-Gal*f* transferase activity and possesses a novel glycosyltransferase fold (GT111). An overlay of the AlphaFold 3 PamA structural prediction with *K. pneumoniae* WbbM revealed structural similarity with the GT111 N-terminal domain (Fig. S1*B*). Analysis of PamC revealed that its closest homologs are UDP-Gal*f* glycosyltransferases in the GT2 family. PamC is only 22% identical/35% similar at the amino acid level to the well-studied *Mycobacterium tuberculosis* GlfT2 UDP-Gal*f* transferase, which synthesizes the [→5)-β-Gal*f*-(1 → 6)-β-Gal*f*-(1→]_n_ galactan portion of the arabinogalactan cell wall component (12, 13, 14), but an overlay of the AlphaFold three structural prediction of PamC with GlfT2 demonstrated structural similarity (Fig. S1*C*). These analyses suggest that PamB generates the UDP-Gal*f* donor substrate through conversion from UDP-Gal*p* and that PamA and PamC are UDP-Gal*f* transferases that promote synthesis of the galactan polymer.

In additional experiments, we examined whether *pamA*, *pamB*, and *pamC* are all required for galactan production in *K. kingae*. As *pamABC* are contained in an operon, we generated in-frame premature stop codon mutants in each of the three genes to eliminate the product of the mutated gene and avoid disruption of normal transcription of the downstream gene(s). In addition, we generated complemented strains by integrating the wild-type version of each gene into a separate region of the chromosome (complementation locus) using a previously generated complementation construct (15). As shown in Figure 1*C*, mutant strains KK01*pamA,* KK01*pamB,* and KK01*pamC* all lacked LPS-associated galactan as assessed by deoxycholic acid (DOC)-PAGE gels with silver staining and by Western blot analysis with anti-galactan antiserum GP-19 (5). Complementation of each gene individually in the respective mutants restored galactan expression (Fig. 1*C*). As a parallel approach, we introduced the same premature stop codon mutations in *pamA*, *pamB*, and *pamC* into plasmid pTrc*-pamABC* and then transformed these constructs into *E. coli* K-12 strain JM109. As shown in Figure 1*D*, we observed no GP-19 reactivity with the *pamC* mutant, indicating that *pamC* is absolutely required for galactan production. In contrast, with the *pamA* and *pamB* mutants we observed faint GP-19 reactivity (Fig. 1*D*). *E. coli* JM109 contains an intact chromosomally encoded UDP-Gal*f* mutase called UGM (PamB homolog) in its O-antigen synthesis locus (10), explaining why UDP-Gal*f* is still present to some degree in JM109 p*ABC-*Δ*pamB.* The persistence of some galactan in strain JM109 p*ABC-*Δ*pamA* suggests that PamA supports, but is not essential for, galactan synthesis in *E. coli*. Together, these results indicate that *pamA*, *pamB,* and *pamC* are all essential for galactan production in *K. kingae* but that only PamC is essential for galactan production in *E. coli* JM109.

### Two pamC alleles exist in the *K. kingae* population and are the determinants of galactan structure

**Figure 2:**
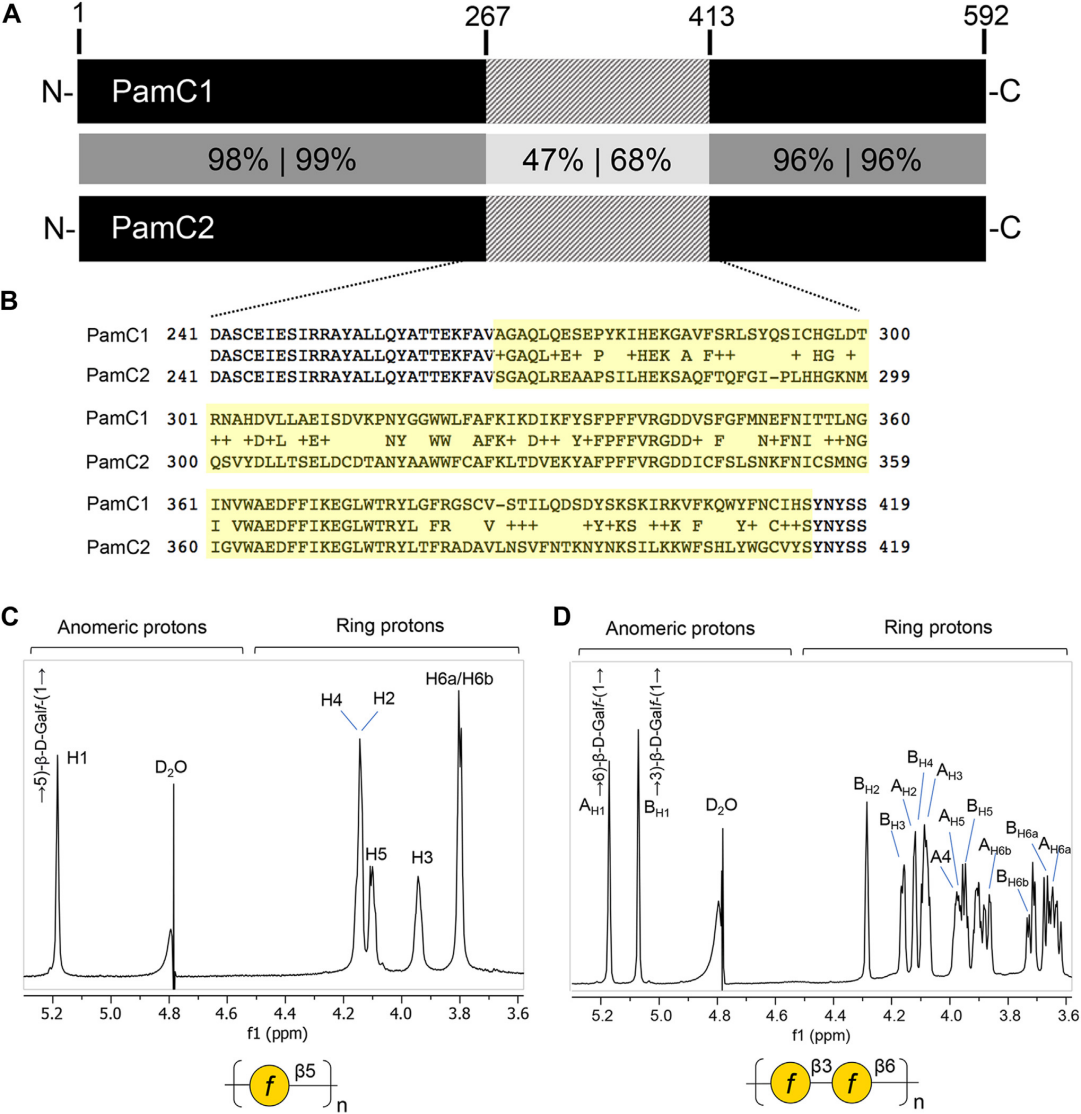
**Two *pamC* alleles (*pamC1* and *pamC2*) are present in *K. kingae* and associate with galactan structure.***A, schematic* representation of the PamC1 and PamC2 proteins from strains KK01 and PYKK181, respectively. The high identity/similarity scores between amino acids 1 to 266 and 414 to 592 are shown in *dark grey boxes*, and the region of lower similarity between amino acids 267 to 413 is shown in a *light grey box*. *B,* amino acid sequence alignment of the 146-residue stretch that constitutes the largest region of dissimilarity between PamC1 and PamC2. *C* and *D,*^1^H NMR spectrum of galactan purified from the surface of *pamC1-*containing strain PYKK58 using 16 scans (*C*) and galactan purified from *pamC2-*containing strain PYKK143 using 16 scans (*D*). The chemical shift assignments for the PYKK58 galactan are in Table S1, and the assignments for the PYKK143 galactan are in Table S2.

Our mutation data in combination with the structural predictions suggest that PamC is directly involved in galactan synthesis, possibly as the galactan synthase. In considering that some strains produce galactan with the structure of [→5)-β-Gal*f*-(1→]_n_ (1) and other strains produce galactan with the structure of [→3)-β-Gal*f*-(1 → 6)-β-Gal*f*-(1→]_n_ (2), we wondered whether the PamC protein varies among strains. Examination of *pamC* sequences in diverse strains in the *K. kingae* population revealed two distinct *pamC* alleles that we designated *pamC1* and *pamC2*, with 85% identity and 90% similarity (Fig. S3). Among strains that possess *pamC1*, the predicted inter-strain amino acid identity of PamC1 is ≥ 98%. Similarly, among strains that possess *pamC2*, the predicted inter-strain amino acid identity of PamC2 is ≥98%. The dissimilarity between PamC1 and PamC2 is contained in an internal 146-amino acid section (Fig. 2, *A* and *B*). The *pamC1* allele is present in our prototype strain KK01, which produces the [→5)-β-Gal*f*-(1→]_n_ galactan (1), and the *pamC2* allele is present in strain PYKK181, which produces the [→3)-β-Gal*f*-(1 → 6)-β-Gal*f*-(1→]_n_ galactan (2). To assess whether there is a correlation between *pamC* allele and galactan type, we performed ^1^H NMR spectroscopic experiments on galactan isolated from an additional *K. kingae* strain that contains the *pamC1* allele (PYKK58, Fig. 2*C* and Table S1) and an additional strain that contains the *pamC2* allele (PYKK143, Fig. 2*D* and Table S2). Strain PYKK58 produced the [→5)-β-Gal*f*-(1→]_n_ galactan, and strain PYKK143 produced the [→3)-β-Gal*f*-(1 → 6)-β-Gal*f*-(1→]_n_ galactan. We will refer to the [→5)-β-Gal*f*-(1→]_n_ galactan as type 1 and the [→3)-β-Gal*f*-(1 → 6)-β-Gal*f*-(1→]_n_ galactan as type 2.

**Figure 3:**
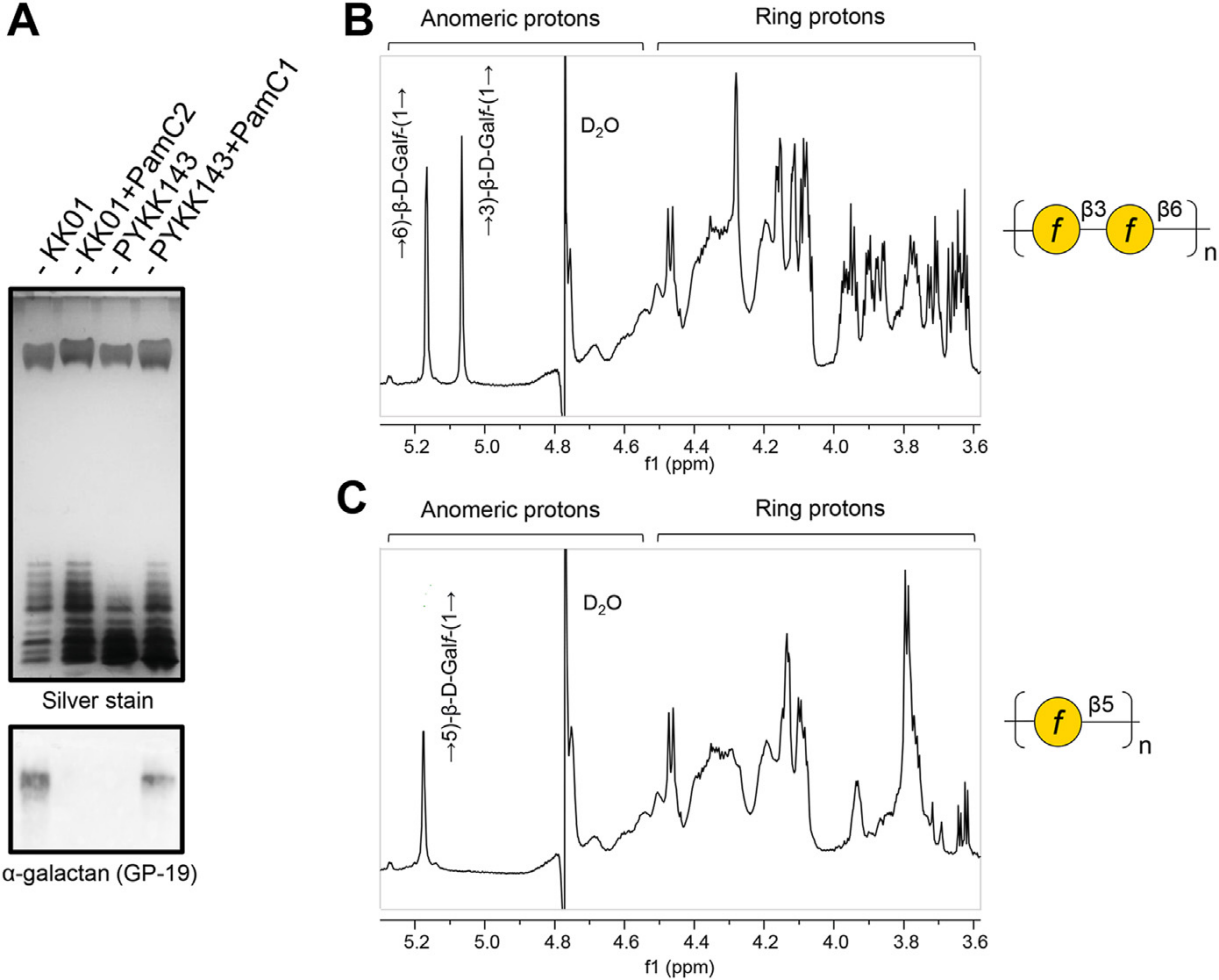
***pamC* allele is the determinant of galactan structure.***A*, *top panel*, silver-stained DOC-PAGE profiles of LPS isolated from strains KK01 (*pamC1* allele), KK143 (*pamC2* allele), and their isogenic mutants KK01+PamC2 (*pamC2* allele) and PYKK143+PamC1 (*pamC1* allele) engineered to express their non-native *pamC* allele. *A*, *bottom panel*, Western blot analysis of the same LPS samples as the *top pane*l, demonstrating that the type 1-specific galactan antiserum GP-19 only reacts with the HMW LPS species from strains containing the *pamC1* allele (KK01 and KK143+PamC1). *B,*^1^H NMR spectrum of LPS isolated from strain KK01+PamC2 (80 scans) demonstrating the presence of β-(1 → 3)-linked and β-(1 → 6)-linked Gal*f*. *C,*^1^H NMR spectrum of LPS isolated from strain PYKK143+PamC1 (160 scans) demonstrating the presence of β-(1 → 5)-linked Gal*f*.

As an additional approach to establish the relationship between *pamC* allele and galactan type, we generated isogenic mutants containing either *pamC1* or *pamC2.* In particular, we replaced the native *pamC1* allele in strain KK01 with the *pamC2* allele, generating strain KK01+PamC2, and we replaced the native *pamC2* allele in strain PYKK143 with the *pamC1* allele, generating strain PYKK143+PamC1. As shown in Figure 3*A*, LPS preparations from both KK01+PamC2 and PYKK143+PamC1 contained galactan as assessed by DOC-PAGE and silver staining, but only LPS from the strains containing *pamC1* (KK01 and PYKK143+PamC1) was reactive with GP-19 antiserum (Fig. 3*A*, bottom panel), which was generated against a type 1 galactan glycoconjugate (5) and is specific for the type 1 galactan. ^1^H NMR analysis confirmed the presence of two anomeric signals due to (1 → 3)-substituted and (1 → 6)-substituted β-Gal*f* in the strain KK01+PamC2, consistent with the production of the type 2 galactan (Fig. 3*B*). The strain PYKK143+PamC1 showed only one anomeric signal due to (1 → 5)-substituted β-Gal*f*, consistent with the production of type 1 galactan (Fig. 3*C*). These data demonstrate that *pamC1* is the determinant of type 1 galactan structure, and *pamC2* is the determinant of type 2 galactan structure. In addition, these results suggest that PamC1 and PamC2 are directly involved in generating the type 1 and type 2 galactan, respectively, potentially as synthases that generate the galactan polymer.

### PamC1 and PamC2 are sufficient for galactan synthesis *in vitro*

**Figure 4:**
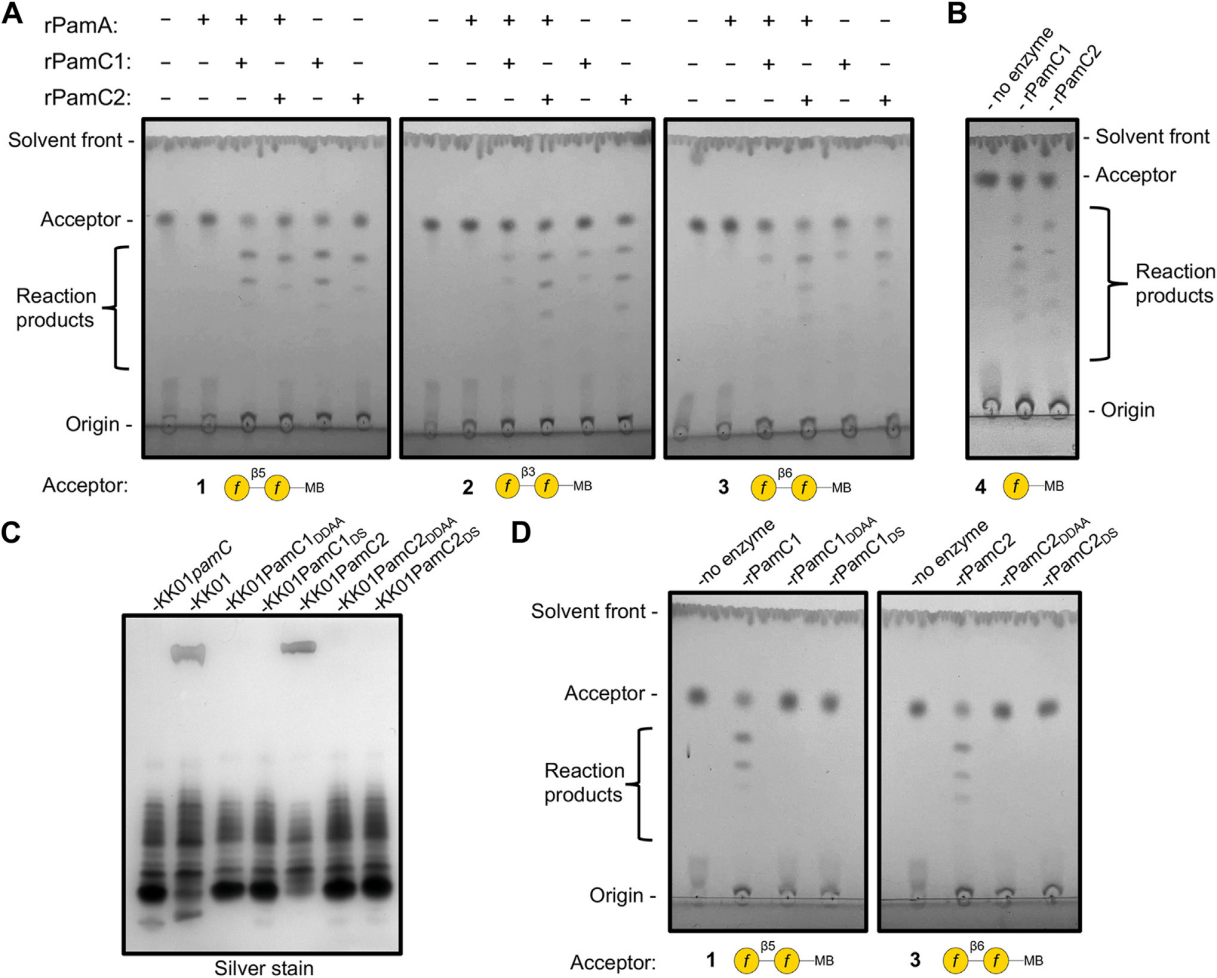
**PamC1 and PamC2 are UDP-Gal*f* transferases capable of synthesizing the galactan homopolymer.***A*, thin layer chromatography (TLC) separations of synthetic Gal*f* disaccharide acceptors modified by recombinant PamA (rPamA), PamC1 (rPamC1), and PamC2 (rPamC2). Acceptors **1** (*left panel*), **2** (*middle panel*), and **3** (*right panel*) were incubated with UDP-Gal*f* in the presence or absence of the noted recombinant proteins, and aliquots were subjected to TLC separation and visualization of the reaction products. *B*, acceptor **4** was incubated with UDP-Gal*f* in the presence or absence of rPamC1 or rPamC2, and aliquots were subjected to TLC separation and visualization of the reaction products. *C*, LPS profiles of PamC1 and PamC2 critical site mutants. LPS was isolated from strains KK01*pamC*, KK01, KK01+PamC1_D239A/D241A_ (KK01+PamC1_DDAA_), KK01+PamC1_D343S_ (KK01+PamC1_DS_), KK01+PamC2, KK01+PamC2_D239A/D241A_ (KK01+PamC2_DDAA_), and KK01+PamC2_D343S_ (KK01+PamC2_DS_), separated using DOC-PAGE, and stained with silver nitrate. *D,* TLC separations of Gal*f* disaccharide acceptors modified by recombinant PamC1 and PamC2 mutated for the predicted critical site residues. rPamC1, rPamC1_D239A/D241A_ (rPamC1_DDAA_), and rPamC1_D343S_ (rPamC1_DS_) were incubated with UDP-Gal*f* and **1**. rPamC2, rPamC2_D239A/D241A_ (rPamC2_DDAA_), and rPamC2_D343S_ (rPamC2_DS_) were incubated with UDP-Gal*f* and **3**. MB, *p-*methoxybenzamide tag.

To test the hypothesis that PamC1 and PamC2 directly synthesize the galactan homopolymer, we assessed their ability to utilize UDP-Gal*f* as an activated donor sugar to extend synthetic Gal*f* acceptors (**1**–**4**) (Fig. S4) with additional Gal*f* residues *in vitro* using thin layer chromatography (TLC). *p-*Methoxybenzamide-tagged disaccharide acceptors with non-reducing terminal β-(1 → 5)- (**1**), β-(1 → 3)- (**2**), or β-(1 → 6)-linked Gal*f* residues (**3**) and purified recombinant PamC1 (rPamC1) and PamC2 (rPamC2) were used in this analysis (16). As shown in Figure 4*A*, both rPamC1 and rPamC2 were able to extend the acceptors with multiple Gal*f* residues, regardless of the linkage of the non-reducing terminal Gal*f* of the acceptor. The addition of Gal*f* residues was dependent on the presence of a PamC protein. Given the observed substrate flexibility of rPamC1 and rPamC2, we next examined whether the enzymes extend a Gal*f* monosaccharide acceptor (**4**). As shown in Figure 4*B*, both enzymes were able to extend **4**, indicating that a single non-reducing terminal Gal*f* residue is sufficient as an acceptor for rPamC1 and rPamC2 under these *in vitro* conditions. These results indicate that PamC1 and PamC2 are galactan synthases capable of generating Galf homopolymers on synthetic Galf disaccharide and monosaccharide acceptors in a process that does not require PamA.

### Predicted critical site residues are essential for PamC1 and PamC2 function

Given the similarity between PamC1 and PamC2 and GlfT2 based on structural predictions, we hypothesized that motifs that are critical for enzyme function in GlfT2 are also present in PamC1 and PamC2. GlfT2 contains a DXD motif that has been shown to be critical for binding to activated-sugar donors as well as a downstream DD motif that is directly involved in the catalytic mechanism (12). The DXD and DD motifs are conserved in PamC1 and PamC2 and are located at amino acids 239 to 241 (DXD motif) and amino acids 342 and 343 (DD motif) (Fig. S5). Using site-directed mutagenesis, we introduced unmarked D239A/D241A mutations and a D343S mutation separately into PamC1 of strain KK01 and PamC2 of strain KK01+PamC2. Consistent with our hypothesis, DOC-PAGE separation combined with silver staining of LPS from these mutants revealed the absence of the galactan (Fig. 4*C*). Recombinant PamC1_D239A/D241A_, PamC1_D343S_, PamC2_D239A/D241A_, and PamC2_D343S_ were purified and examined for their ability to extend the previously described synthetic Gal*f* acceptors. As shown in Figure 4*D*, both mutations abrogated the ability of both PamC1 and PamC2 to extend **1** and **3**, respectively, with additional Gal*f* residues. Taken together, the *K. kingae* mutational data and the data with purified recombinant proteins demonstrate that the DXD and DD motifs are critical for the PamC1 and PamC2 galactan synthase function.

### PamC1 is a monofunctional UDP-Galf transferase, and PamC2 is a bifunctional UDP-Galf transferase

**Figure 5:**
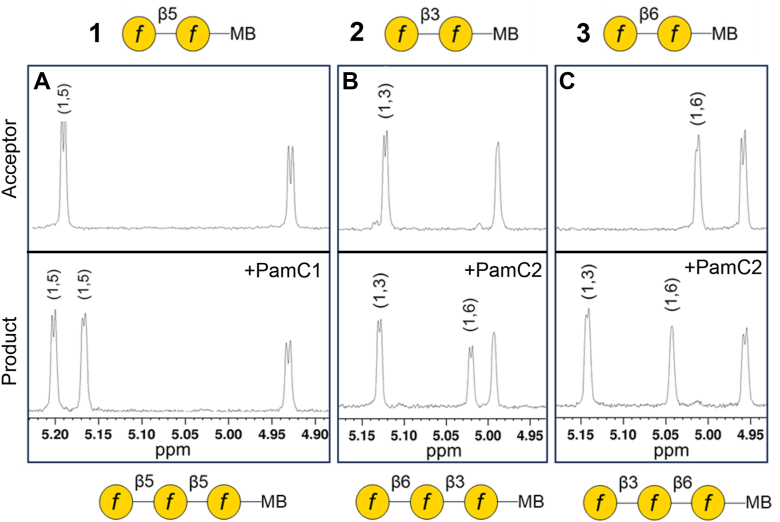
**PamC1 creates a β-(1** → **5) Gal*f* linkage and PamC2 creates both β-(1** → **3) and β-(1** → **6) Gal*f* linkages.**^1^H NMR spectroscopy was used to investigate the anomeric proton chemical shifts of synthetic *p-*methoxybenzamide-tagged Gal*f* disaccharide acceptors (*top* spectra) and the same acceptors modified to Gal*f* trisaccharides through *in vitro* rPamC1 or rPamC2 modification (*bottom* spectra). *A*, **1** was modified by rPamC1. *B*, **2** was modified by rPamC2. C, **3** was modified by rPamC2. The structures of the acceptors (*above* spectra) and the products (*below* spectra) are shown. The anomeric peak furthest to the *right* for each spectrum is the H-1 signal. For the *top* spectra, the peak on the *left* is the H-1′ signal. The *bottom* spectra contain an additional peak representing the H-1″ signal from the PamC1 or PamC2-modified acceptors. MB, *p-*methoxybenzamide tag.

We next determined the linkages of the terminal residues of the Gal*f* trisaccharides (+1 Gal*f* products) that were generated following modification of the synthetic Gal*f* disaccharide acceptors by rPamC1 or rPamC2 in the presence of UDP-Gal*f*. The trisaccharide products from **1** modified by rPamC1, **2** modified by rPamC2, and **3** modified by rPamC2 were analyzed using ^1^H NMR spectroscopy. The ^1^H NMR spectrum for the PamC1-modified **1** (Fig. 5*A*) is consistent with a previously reported synthetic Gal*f* trisaccharide acceptor (containing an octyl aglycon instead of a *p-*methoxybenzamide-octyl tag) containing an additional non-reducing terminal β-(1 → 5)-Gal*f* residue (17), indicating that PamC1 generates a β-(1 → 5) linkage. The ^1^H NMR spectrum for the PamC2-modified **2** (Fig. 5*B*) is consistent with addition of a β-(1 → 6)-linked Gal*f*, and the spectrum for the PamC2-modified **3** (Fig. 5*C*) is consistent with addition of a β-(1 → 3)-linked Gal*f*, based on identical molecules that were chemically synthesized and characterized previously (16). These results indicate that PamC1 is a monofunctional galactan synthase capable of creating a β-(1 → 5) Gal*f* linkage while PamC2 is a bifunctional galactan synthase capable of creating β-(1 → 3) and β-(1 → 6) Gal*f* linkages.

## Discussion

*K. kingae* produces an atypical LPS that contains a galactofuranose homopolymer called galactan. In this study, we investigated the molecular basis of galactan synthesis. Mutational studies in *K. kingae* demonstrated that the predicted PamA and PamC UDP-Gal*f* transferases and the predicted PamB UDP-Gal*p* mutase are essential for galactan production. Bioinformatic analysis revealed that two *pamC* alleles, referred to as *pamC1* and *pamC2*, exist in the *K. kingae* population, and NMR analysis revealed a correlation between *pamC* allele and galactan structure. Analyses of isogenic *pamC* allele swap strains established that *pamC1* is the determinant of the type 1 galactan structure ([→5)-β-Gal*f*-(1→]_n_) and *pamC2* is the determinant of the type 2 galactan structure ([→3)-β-Gal*f*-(1 → 6)-β-Gal*f*-(1→]_n_). In an *in vitro* system with synthetic Gal*f* disaccharide acceptors, recombinant PamC1 and PamC2 were capable of extending the acceptors with multiple Gal*f* residues, confirming that they are galactan synthases. Predicted critical sites were identified through homology analysis and were confirmed to be essential for PamC1 and PamC2 function in *K. kingae* and *in vitro*. Finally, structural analysis of PamC1- and PamC2-modified acceptors demonstrated that PamC1 is a monofunctional enzyme that generates β-(1 → 5)-linked Gal*f* and PamC2 is a bifunctional enzyme that generates β-(1 → 3) and β-(1 → 6) Gal*f* linkages.

Bioinformatic analysis and structural predictions indicate that GT2 family UDP-Gal*f* transferases exist in diverse bacteria and are associated with a variety of different Gal*f* homopolymers containing different linkages (16). The most well-studied member of this group is the *M. tuberculosis* GlfT2 enzyme, a bifunctional processive enzyme that contains a single active site capable of creating alternating β-(1 → 5) and β-(1 → 6) Gal*f* linkages (12, 13, 14). Of the other three biochemically characterized UDP-Gal*f* transferases in this family, two are bifunctional. Specifically, both the *K. kingae* PamC2 protein and the *Salmonella enterica* serovar Typhimurium LT2 strain SL3770 STM0724 protein generate β-(1 → 3) and β-(1 → 6) linkages. However, STM0724 appears to possess relaxed fidelity compared to PamC2, as evidenced by the presence of both [→3)-β-Gal*f*-(1 → 6)-β-Gal*f*-(1→]_n_ disaccharide and [→3)-β-Gal*f*-(1 → 3)-β-Gal*f*-(1 → 6)-β-Gal*f*-(1→]_n_ trisaccharide repeat units in the T1 antigen structure synthesized by this enzyme (16). PamC1 is the only characterized enzyme in this family that has monofunctional activity in whole bacteria, creating only β-(1 → 5) Gal*f* linkages. However, analysis by Kelly *et al.* (2024) identified multiple other bacterial protein orthologs that produce a galactofuranose homopolymer, including many examples with a single linkage (including β-(1 → 2)-linked and β-(1 → 6)-linked galactans) (16). Further detailed biochemical and structural analyses will be necessary to gain insight into the mechanistic differences that dictate monofunctional *versus* bifunctional galactosyltransferase activity in this enzyme family. *K. kingae* PamC1 and PamC2 are attractive representatives for this analysis, as these two proteins have highly homologous amino acid sequences aside from a 146-amino acid stretch with significant dissimilarity that is likely responsible for monofunctional *versus* bifunctional enzyme activity.

In our *in vitro* studies, PamC1 and PamC2 displayed relaxed substrate specificity. All three of the disaccharide acceptors were extended by both enzymes (Fig. 4*A*), suggesting that the existing linkage of the terminal non-reducing Gal*f* residue of the acceptor is not a critical factor in dictating the ability of PamC1 and PamC2 to synthesize galactan homopolymer. Indeed, a monosaccharide Gal*f-*containing acceptor was also able to be extended by PamC1 and PamC2 (Fig. 4*B*). This observation is similar to observations *in vitro* with recombinant GlfT2, where a different monosaccharide Gal*f-*containing aglycon acceptor was able to be extended with up to 46 Gal*f* residues (18). Kinetic analysis of GlfT2 by Rose *et al.* (19) found that while this enzyme can extend both disaccharide and trisaccharide Gal*f* acceptors, the enzyme displays a greater affinity for the trisaccharide acceptors. The artificial *in vitro* conditions in which the relaxed specificities have been observed could be forcing or overriding the selectivity of these enzymes *in vivo*, where a stricter specificity is maintained in the presence of their larger, natural substrates.

In this study, we were unable to define the role of PamA, a predicted GT111 UDP-Gal*f* transferase that is encoded in the *pamABC* operon. The only characterized member of this newly described glycosyltransferase family is the N-terminal domain of the bifunctional O-antigen synthase WbbM of *K. pneumoniae* serotype O2a (11). The N-terminal domain of WbbM is a monofunctional UDP-Gal*f* transferase, and the C-terminal domain of WbbM is a monofunctional GT8 family UDP-Gal*p* transferase. Together, WbbM synthesizes the [→3)-α-Gal*p*-(1 → 3)-β-Gal*f*-(1→]_n_ serotype O2a O-antigen (11). In contrast, PamA consists of only one predicted glycosyltransferase fold (Fig. S1*B*) and is not necessary for galactan synthesis when an appropriate non-reducing terminal Gal*f*-containing acceptor is present. One logical hypothesis for the role of PamA is that it adds the first Gal*f* residue to the acceptor on which the galactan is built. In studied systems, the O-antigen is synthesized on an undecaprenyl phosphate (Und-P) acceptor on the cytoplasmic side of the inner membrane (20). Synthesis and transport to the periplasm of the acceptor-O-antigen complex is accomplished *via* one of three pathways (Wzy-dependent, ABC transporter-dependent, or synthase-dependent) where the O-antigen is then transferred to the LPS by a ligase enzyme either as repeat units for the Wzy-dependent pathway or as a whole for the ABC transporter-dependent and synthase-dependent pathways (see (21) for a review of O-antigen biosynthesis). Prior to initiation of O-antigen synthesis, an initiating transferase adds a nucleotide diphosphate sugar to Und-P to generate Und-P-P-sugar, on which the dedicated O-antigen glycosyltransferase(s) build the polymer (6, 21). Classical O-antigen systems typically encode at least one dedicated glycosyltransferase that acts on the Und-P-P-sugar to add the appropriate sugar (or sugars) to the acceptor to prime O-antigen synthesis. This is necessary as the synthase enzyme(s) requires an acceptor containing a non-reducing terminal sugar residue that is the same as the residue that constitutes the polymer in homopolymeric O-antigens or is one of the residues in the repeating unit of a heteropolymeric O-antigen. Future work will explore the hypothesis that PamA serves this critical role in priming galactan synthesis in *K. kingae*.

The presence of only two galactan structures in the *K. kingae* population is reminiscent of the limited number of capsular polysaccharide types (four) in this species (22, 23). The sequenced *K. kingae* isolates available in the NCBI database represent a diverse collection of isolates from both invasive infections and healthy carriers (24). While the galactan structures from only four strains have been structurally characterized to date, the fact that there are only two *pamC* alleles in the sequenced collection indicates that there are only two galactan structures in this species, contrasting with species such as *E. coli*, which has >170 O-antigen types (25). In the case of *K. kingae*, two capsule types (type a and b) are represented in >95% of invasive disease isolates, while types c and d have mostly been identified in carriage isolates (22, 23). It is not currently known whether similar correlations with invasive disease *versus* carriage exist in terms of galactan type. In addition to the galactan, *K. kingae* LPS also appears to be extended with a more traditional uncharacterized O-antigen, as evidenced by appearance of a laddering pattern when the LPS is resolved using electrophoresis (see Figs. 1*C* and 3*A*, and 4*B*), at least in the limited number of strains examined to date. How widespread and how diverse the O-antigen structure is in *K. kingae* is currently unknown. However, previous work has demonstrated that *pamD* and *pamE,* the two genes predicted to encode glycosyltransferases immediately downstream of *pamABC*, are essential for LPS to be modified with the O-antigen (5). The presence of both *pamD* and *pamE* is ubiquitous in the *K. kingae* population, suggesting that the O-antigen is widespread in this species, similar to galactan.

In this work, we identified the synthase responsible for the biogenesis of the *K. kingae* galactan, a key virulence determinant in this organism. One monofunctional version and one bifunctional version of the synthase were identified and were biochemically demonstrated to generate different galactan structures. Future work will explore how galactan synthesis ties into LPS biogenesis and how galactan type impacts pathogenicity in this species.

## Experimental procedures

### Bacterial strains and culture conditions

*K. kingae* strains were cultured on chocolate agar in a humidified atmosphere at 37 °C supplemented with 5% CO_2_ for 16 to 20 h. *E. coli* strains were cultured on LB agar in a humidified atmosphere at 37 °C or in LB broth shaking at 250 rpm at 37 °C with 100 μg/ml ampicillin or 50 μg/ml kanamycin, as appropriate. All *K. kingae* strains, *E. coli* strains, and plasmids used in this study are listed in Table S3. *K. kingae* and *E. coli* strains were stored at −80 °C in brain heart infusion (BHI) containing 20% glycerol or LB containing 15% glycerol, respectively.

### Molecular biology and strain construction

For molecular cloning, Q5 HiFi Master Mix (New England Biolabs) was used for PCR. All restriction enzymes and Gibson Assembly Master Mix were sourced from New England Biolabs. Site-directed mutagenesis was accomplished using the Agilent QuikChange Lightning Kit (Agilent). All plasmids were confirmed to be correct through a combination of restriction digest, PCR, and Sanger sequencing. The primers used in this study are listed in Table S4.

*K. kingae* strain KK01*csaA*, a stable derivative of septic arthritis clinical isolate 269 to 492 that expresses the type 1 galactan and contains an unmarked deletion of its native capsule synthesis locus (5, 26), was used as the strain background for most of the work in this study. For the *pamC* allele swap studies, the *pamC2-*encoding strain PYKK143 was used due to its superior transformability over other strains that encode *pamC2*. A non-encapsulated derivative (PYKK143*csb*) was generated as previously described (22) by introducing the capsule synthesis locus deletion construct pSwapEmpty *via* natural transformation and screening transformants for a loss of capsule expression.

#### *Generation of in-frame pamA, pamB, and pamC nonsense mutants and complements.*

To generate in-frame nonsense mutants of *pamA*, *pamB*, and *pamC*, we first generated a kanamycin-marked construct containing ∼1000 bp of sequence upstream of *pamA*, a kanamycin resistance cassette upstream of the *pamA* promoter, and the *pamABC* genes. The fragment containing ∼1000 bp upstream of *pamA* was amplified from strain KK01 with primers pamAupF/pamAupR and was ligated into EcoRI/KpnI-digested pUC19, generating p*pamA*up. The fragment containing the promoter of *pamA* through ∼1000 bp downstream of the *pamC* stop codon was amplified with primers pamABC_markedF/pamABC_markedR and was ligated into BamHI/SalI-digested p*pamA*up, generating p*pamABC*. The *aphA3* kanamycin resistance cassette was amplified from plasmid pFalcon2 with primers aphA3_KpnIF/aphA3_BamHI and was ligated into KpnI/BamHI-digested p*pamABC,* generating pKan-*pamABC*. To introduce point mutations resulting in the generation of stop codons into the open reading frames of *pamA*, *pamB*, and *pamC*, plasmid pKan-*pamABC* was individually subjected to site-directed mutagenesis with primers pamA mut F/pamA mut R, pamB mut F/pamB mut R, and pamC mut F/pamC mut R, generating plasmids pKan-*pamA*stop, pKan-*pamB*stop, and pKan-*pamC*stop, respectively. The same site-directed mutagenesis primer pairs were used to separately introduce the nonsense mutations into plasmid pTrc99a-*pamABC,* generating pTrc-*pamABCΔpamA*, pTrc-*pamABCΔpamB*, and pTrc-*pamABCΔpamC*.

To generate genetic complements of the *pamA*, *pamB*, and *pamC* in-frame nonsense mutants, we used plasmid pComp-Erm, which has been used successfully to chromosomally complement gene deletions and mutations in *K. kingae* (15). The *pamA* ORF with its native promoter was amplified from strain KK01 using primers pamA comp F/pamA comp R and was ligated into KpnI/BamHI-digested pComp-Erm, generating pComp-*pamA*. As *pamB* and *pamC* are under the control of the *pamA* promoter, we used a derivative of pComp-Erm containing the *K. kingae pilA1* promoter (pComp_*pilA1*_). The ORFs of *pamB* and *pamC* starting at the predicted start codon, were separately amplified from KK01 and were ligated into KpnI/XbaI-digested pComp_*pilA1*_ for *pamB* and KpnI/BamHI-digested pComp_*pilA1*_ for *pamC*, generating pComp_*pilA1*_-*pamB* and pComp_*pilA1*_-*pamC*, respectively.

To introduce the point mutations into *K. kingae*, the plasmids pKan-*pamA*stop, pKan-*pamB*stop, and pKan-*pamC*stop were linearized and transformed into strain KK01*csaA via* natural transformation as previously described (15, 27), generating strains KK01*pamA*, KK01*pamB*, and KK01*pamC*, respectively. The respective mutated gene from each strain was PCR amplified and confirmed to contain the correct nonsense mutations by Sanger sequencing. The complementation plasmids were linearized and introduced to their respective mutant strain *via* natural transformation. While the *aphA3* cassette was placed upstream of the predicted *pamA* promoter, we noticed that a strain harboring this marker but possessing an otherwise wild-type *pamABC* locus had reduced LPS-associated galactan, likely due to reduced transcription of the *pamABC* locus. To remedy this defect, the *aphA3* cassette was removed from the chromosome from the stop codon mutants and their respective complemented strains. This was accomplished by subjecting each strain to the spot transformation procedure (22) with an DNA fragment containing ∼500 bp sequence on either side of the *aphA3* insertion site (amplified from KK01 with primers pam_unmark F/pam_unmark R) and screening transformants for loss of kanamycin resistance and retention of the previously introduced nonsense mutations with Sanger sequencing.

#### *Generation of pamC allele swap strains and critical site point mutants.*

A multi-step cloning process was used to generate the *pamC* allele swap strains. First, using KK01 as the template, primers pamC1Swap_F/pamC1Swap_R were used to amplify a region consisting of ∼30 bp upstream of the predicted *pamC1* start codon to ∼30 bp downstream of the predicted *pamD* stop codon. This fragment was ligated into SalI/KpnI-digest pUC19, generating plasmid p01*pamCD*. The plasmid pKan-*pamABC* was transformed into KK01*csaA* to generate strain KK01*csaA*mark*pamA*. Genomic DNA was isolated from this strain and used as the template with primers pamAupF/pamB_R1 to amplify a fragment starting ∼1000 bp upstream of the kanamycin resistance marker to ∼30 bp downstream of *pamB*. This fragment was ligated into EcoRI/SalI-digested p01*pamCD*, generating plasmid p01Swap, which contains a kanamycin resistance cassette upstream of *pamA* and a SalI site in the ∼70 bp intergenic region between *pamB* and *pamC*. This plasmid was used to transform strain PYKK143*csb* to generate strain PYKK143+PamC1.

To generate a similar swap plasmid containing *pamC2* from strain PYKK181, a Gibson assembly approach was used. Primers pamC2Swap_F/pamC2Swap_R were designed using NEBuilder (New England Biolabs) to amplify a fragment with an introduced SalI site in the same location upstream of *pamC2* from PYKK181 as was generated in the construction of p01Swap. This fragment was then assembled into the SalI/PstI backbone fragment of p01Swap using Gibson Assembly Master Mix, generating plasmid p181Swap. This plasmid was used to transform strain KK01*csaA* to generate strain KK01+PamC2. After genotyping confirmation, the kanamycin resistance cassette was removed from strains KK01+PamC2 and PYKK143+PamC1 as described above.

To introduce *pamC1* and *pamC2* critical site point mutants into *K. kingae*, the primers pamC_DDD_sense/pamC_DDD_anti were used to introduce the D239A/D241A mutations to both p01Swap and p181Swap, generating plasmids p01Swap_D239A/D241A_ and p181Swap_D239A/D241A_, respectively. The primers pamC1_D343S_sense/pamC1_D343S_anti and pamC2_D343S_sense/pamC2_D343S_anti were used to introduce the D343S mutation to p01Swap and p181Swap, generating plasmids p01Swap_D343S_ and p181Swap_D343S_, respectively. The plasmids were introduced in strain KK01*csa*A *via* natural transformation, and the transformants were screened for incorporation of the mutant *pamC1* or *pamC2* allele using PCR and Sanger sequencing, resulting in strains KK01+PamC1_D239A/D241A_, KK01+PamC1_D343S_, KK01+PamC2_D239A/D241A_, and KK01+PamC2_D343S_. The kanamycin resistance cassette upstream of *pamA* was then removed as described above.

##### Generation of recombinant protein expression constructs

To generate constructs for the expression of recombinant PamA, PamB, PamC1, and PamC2 with an N-terminal histidine affinity tag (HAT), each ORF was individually amplified from strain KK01 (PamA, PamB, and PamC1) or PYKK181 (PamC2) starting at the predicted start codon with primers PamA_HAT_F/PamA_HAT_R, PamB_HAT_F/PamB_HAT_R, and PamC_HAT_F/PamC_HAT_R and were ligated into BamHI/EcoRI-digested pHAT10, generating plasmids pHAT-PamA, pHAT-PamB, and pHAT-PamC1 and pHAT-PamC2. The primers pamC_DDD_sense/pamC_DDD_anti were used to introduce the D239A/D241A mutations to both pHAT-PamC and pHAT-PamC2, and the primers pamC1_D343S_sense/pamC1_D343S_anti and pamC2_D343S_sense/pamC2_D343S_anti were used to introduce the D343S mutation to pHAT-*pamC1* and pHAT-*pamC2*, respectively.

#### Galactan purification

Milligram quantities of galactan were purified using the method described in the Supplementary Materials and Methods of Montoya *et al.* (5). The galactan was isolated from strains PYKK58*csb* (22) and PYKK143*csb* (this study), which are deleted for their capsule synthesis loci to eliminate the possibility of contaminating capsular polysaccharide signals in downstream NMR analysis.

#### LPS isolation

LPS was prepared as previously described (5). Briefly, bacterial lawns from overnight growth were collected by swabbing from agar plates into phosphate buffered saline (PBS). The resulting pellet was resuspended in endotoxin-free water and an equal volume of 90% phenol. After agitating at 70 °C for 1 h, the mixture was centrifuged at 10,000*g* for 10 min, and the aqueous phase was saved, the phenolic phase was extracted two more times, and the aqueous phases were pooled and washed twice with diethyl ether to remove residual phenol. The samples were then flash frozen and lyophilized, resuspended in PBS supplemented with 2.5 mM MgCl_2_ and 0.1 mM CaCl_2_, and treated with 2 units DNase I and 100 μg/ml RNase A for 6 h at 37 °C and 100 μg/ml proteinase K at 45 °C for 16 to 20 h. The samples were dialyzed extensively against water and lyophilized to determine dry weight. Samples were stored at room temperature lyophilized or at 5 mg/ml in water at −20 °C.

#### DOC-PAGE, silver stain, and Western blot analyses

LPS samples were boiled for 5 min and separated using polyacrylamide gels containing deoxycholic acid (DOC) in the gel and running buffer, were fixed overnight in 40% ethanol and 5% acetic acid with at least two fixation solution changes, and were then stained with silver nitrate (28, 29). For Western blotting, the gels were transferred to nitrocellulose, blocked with 5% skim milk powder in PBS, and probed with a 1:1000 dilution of anti-galactan antiserum GP-19 (5). After washing, a 1:5000 dilution of anti-guinea pig-horseradish peroxidase secondary antibody was added, followed by a chemiluminescent peroxidase substrate. The blot and gel images were captured using a G:BOX Chemi XX6 Gel Imaging System (Syngene, Cambridge, UK).

#### Bioinformatic analyses

Primary nucleotide and amino acid sequences were analyzed with nucleotide BLAST (BLASTn) or protein BLAST (BLASTp) hosted at the National Center for Biotechnology Information (www.ncbi.nlm.nih.gov). Pairwise alignments were generated using EMBOSS Needle and multiple sequence alignments were generated using Clustal Omega, both hosted at the European Molecular Biology Lab’s European Bioinformatics Institute (EMBL-EBI) (www.ebi.ac.uk). Structural prediction was accomplished using AlphaFold 3 (9) accessed *via* www.alphafoldserver.com. Structural overlays were generated using the RCSB PDB Pairwise Structure Alignment tool (30) accessed *via* www.rcsb.org/alignment. Structural overlays were visualized using UCSF ChimeraX v1.9 (31).

#### Recombinant protein expression and purification

Plasmids pHAT-*pamA*, pHAT-*pamB*, pHAT-*pamC1,* and *pHAT-pamC2* were individually electroporated into *E. coli* BL21(DE3), and recombinant protein induction and purification conditions were the same for all four proteins. An overnight culture was back diluted 1:100 in 500 ml LB with 100 μg/ml ampicillin and was incubated shaking at 37 °C until the OD_600_ reached ∼0.4. IPTG was added to a final concentration of 0.4 mM, and the culture was induced shaking at 30 °C for 3 h. The resulting bacterial pellet was resuspended in 50 ml binding buffer (50 mM sodium phosphate pH 7.4, 500 mM NaCl, 40 mM imidazole) containing cOmplete EDTA-free Protease Inhibitor Cocktail (MilliporeSigma) and was sonicated 3 × 30 s on 40% amplitude (Q500 sonicator, QSonica). The whole cell sonicate was clarified by centrifugation at 12,000*g* for 30 min at 4 °C before being applied to 5 ml HisTrap column (Cytiva) using an ÄKTA Pure 25L FPLC System (Cytiva). After extensive washing with binding buffer, the bound protein was eluted with binding buffer containing 500 mM imidazole. Fractions containing the recombinant protein were pooled and buffer exchanged into 100 mM potassium phosphate buffer pH 7.4 (Kphos buffer) using Amicon Ultra Centrifugation filters (MilliporeSigma) of appropriate molecular weight cutoffs. The protein concentrations were determined using the Bio-Rad Protein Assay Kit with bovine serum albumin as the standard.

#### PamB *in vitro* activity assay and high-performance liquid chromatography (HPLC)

HPLC was used to detect the conversion of UDP-Gal*f* to UDP-Gal*p* by recombinant PamB as previously described (32) with some modifications. A 200 μl total reaction volume containing 0.5 mM UDP-Gal*f*, 20 mM DTT, 0.75 μM rPamB (or rPamC as a negative control) in 100 mM Kphos buffer was incubated at 37 °C for 1 h and then 90 °C for 5 min to denature the proteins. The samples were then applied to a Amicon Ultra 10,000 MWCO filter, and 2 μl of the flow-through was injected into an Microsorb-MC 100-5 C_18_, 250 x 4.6 mm HPLC column (Agilent) using an Agilent Series 1100 instrument. The reaction products were separated with an isocratic flow of 50 mM triethylammonium acetate (TEAA) pH 6.6 containing 1.5% acetonitrile at a flow rate of 0.6 ml/min with detection at 262 nm. Samples of UDP-Gal*p* and UDP-Gal*f* were run separately as controls.

#### PamC *in vitro* activity assay and thin layer chromatography (TLC)

TLC was used to investigate the ability of recombinant PamC1 and PamC2 to add additional Gal*f* residues onto synthetic *p-*methoxybenzamide-tagged Gal*f* disaccharide and monosaccharide acceptors (**1**–**4**) (Fig. S4). A 20 μl reaction volume containing 1 μM acceptor, 15 μg UDP-Gal*f*, and 2 μM enzyme in 50 mM HEPES pH 7.5, 20 mM MgCl_2_, 2 mM DTT was incubated at 30 °C for 2 h. Three microliters (1 μl at a time with drying between applications) was spotted on silica gel on TLC aluminum foils with fluorescence indicator 254 μM (MilliporeSigma) and air dried before separation with solvent containing 40% butanol, 25% ethyl acetate, 20% water, and 15% acetic acid. After drying, the silica gel foil was exposed to 254 nm UV light and imaged. For reactions that contained two proteins, both were included at 2 μM concentrations each. Controls included reactions with no enzymes.

A scaled-up *in vitro* reaction and preparative TLC separation scheme was used to generate and isolate +1 Gal*f-*modified *p-*methoxybenzamide-tagged Gal*f* disaccharide acceptors (modified to a trisaccharide) for ^1^H NMR analysis. The reaction size was scaled to a total volume of 1 ml and was incubated for 16 h at 30 °C. The reaction was then spotted in a line along six 8 cm-wide silica gel TLC foils for each sample. Following separation and drying, the foils were visualized under UV light and the silica containing the +1Gal*f* reaction product was scraped from the foil and collected (Fig. S6). The silica was then agitated for 30 min at ambient temperature in 40% methanol to elute the modified acceptor. After centrifugation and filtering to remove the silica, the sample was lyophilized. After resuspension in water, the sample was applied to a Sep-Pak Light C18 Cartridge (Waters Corporation) according to the manufacturer’s instructions and eluted with 40% methanol. The sample was then lyophilized and subjected to the ^1^H NMR spectroscopy of synthetic acceptors Method described below.

#### ^1^H NMR spectroscopy of galactan and LPS

For galactan and LPS samples, the dry material was deuterium-exchanged two times with D_2_O. Each exchange was followed by freeze-dying. The final exchange was done with 0.70 ml of deuterium oxide (99.96%) + 0.01 mg/ml sodium trimethylsilylpropanesulfonate (DSS) and used for ^1^H NMR analysis. The ^1^H NMR studies were performed at 25 °C on a Varian Inova 600 MHz or a Bruker Avance III 600 MHz spectrometer, equipped with a cryoprobe, using a standard Presat experiment. The number of scans per sample are noted in the relevant Figure Legend. The data were processed using MestReNova.

#### ^1^H NMR spectroscopy of synthetic acceptors

For the synthetic acceptors, the dry sample was deuterium-exchanged three times with D_2_O. Each exchange was followed by freeze-dying. The final exchange was done with 0.70 ml of deuterium oxide (99.9%) containing 0.05 mM DSS and used for ^1^H NMR spectroscopic analysis. The NMR spectroscopic studies were performed at 25 °C on a Bruker 500 MHz spectrometer with a cryoprobe with 32 scans. The data were processed using Bruker TopSpin 3.7.0.

## Data availability

All the data supporting this work is contained within the manuscript.

## Supporting information

This article contains supporting information (1, 2, 5, 10, 11, 13, 15, 16, 22, 26, 32, 33, 34, 35, 36).

## Conflict of interest

The authors declare that they have no conflicts of interest with the contents of this article.
