# Supplementary material for: Identification and characterization of mono- and bifunctional galactan synthases in the pediatric pathogen Kingella kingae
Source: J Biol Chem. 2025 Jun 6;301(7):110345. doi: 10.1016/j.jbc.2025.110345 (PMC12269835; doi:10.1016/j.jbc.2025.110345)
Supplement: Supporting Information [file mmc1.docx]

**Supporting Information**

**Identification and characterization of mono- and bifunctional galactan synthases in the pediatric pathogen *Kingella kingae***

Eric A. Porsch^1^, Mikel Jason Allas^2,3^, Nina R. Montoya^1,4^, Vanessa L. Muñoz^1,4^, Li Tan^5^, Artur Muszyński^5^, Parastoo Azadi^5^, Stephen N. Hyland^6^, Catherine L. Grimes^6,7^, Tzu-Ting Kao^3^, Todd L. Lowary^2,3,8^, and Joseph W. St. Geme III^1,4^*

^1^Children’s Hospital of Philadelphia, Philadelphia, Pennsylvania, USA

^2^Department of Chemistry, University of Alberta, Edmonton, Alberta, Canada

^3^Institute of Biological Chemistry, Academia Sinica, Nangang, Taipei, Taiwan

^4^Perelman School of Medicine, University of Pennsylvania, Philadelphia, Pennsylvania, USA

^5^Complex Carbohydrate Research Center, University of Georgia, Athens, Georgia, USA

^6^Department of Chemistry and Biochemistry, University of Delaware, Newark, Delaware, USA

^7^Department of Biological Sciences, University of Delaware, Newark, Delaware, USA

^8^Institute of Biochemical Sciences, National Taiwan University, Taipei, Taiwan

Materials included:

- Figures S1 – S6
- Tables S1 – S4
- Supporting Methods
- Supporting References


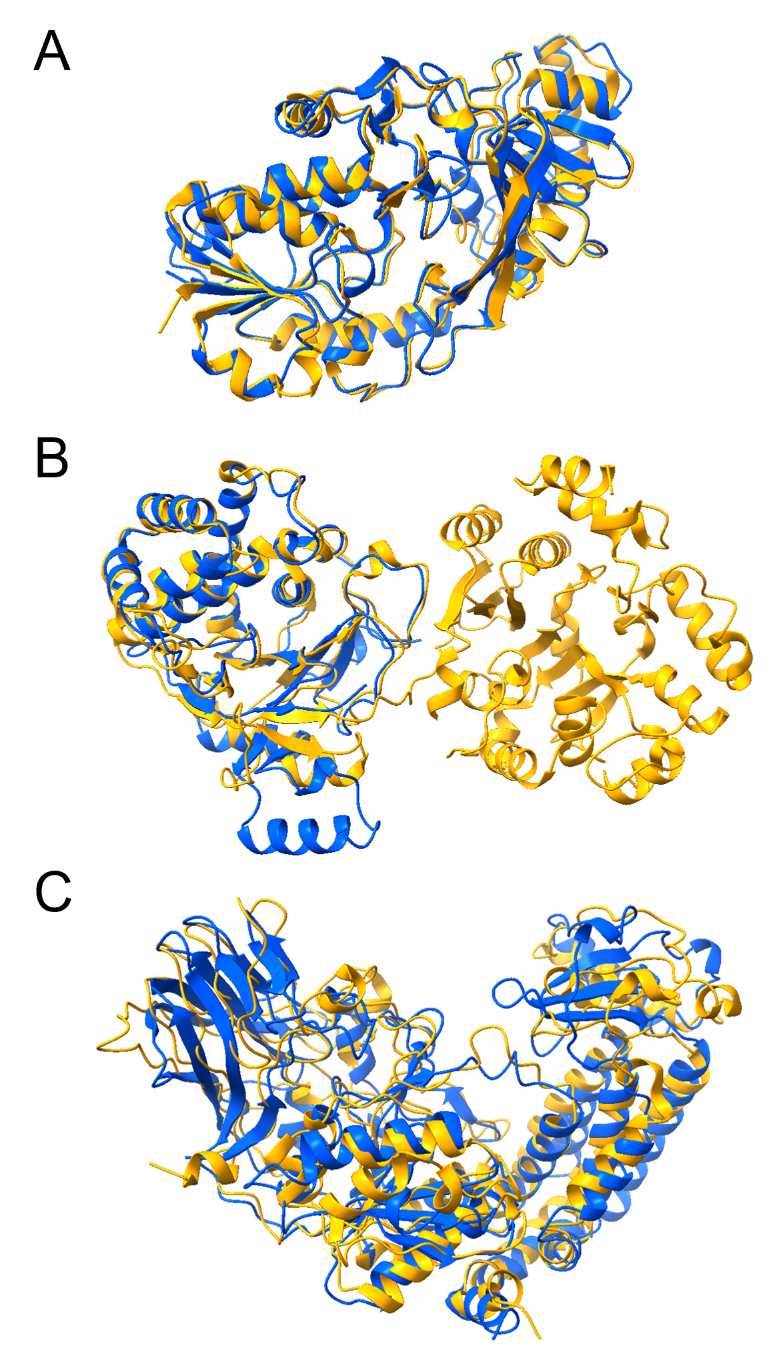


**Figure S1. Structural prediction models of PamB, PamA, and PamC overlaid with crystal structures of characterized proteins containing the same predicted structural folds.** (*A*) The AlphaFold 3 model of PamB (blue, accession AEK99050.1) overlaid with the crystal structure of the *E. coli* UDP-Gal*p* mutase (orange, PDB: 1I8T) (1). (*B*) The AlphaFold 3 model of PamA (blue, accession AEK99049.1) overlaid with the crystal structure of the *K. pneumoniae* serotype O2a WbbM bifunctional glycosyltransferase (orange, PDB: 6U4B) (2). The PamA structural prediction only overlays with the N-terminal GT111 domain of WbbM, not the C-terminal GT8 domain. (*C*) The AlphaFold 3 model of PamC (blue, accession WP_003786867.1) overlaid with the crystal structure of the *M. tuberculosis* GlfT2 UDP-Gal*f* glycosyltransferase (orange, PDB: 4FIX) (3).


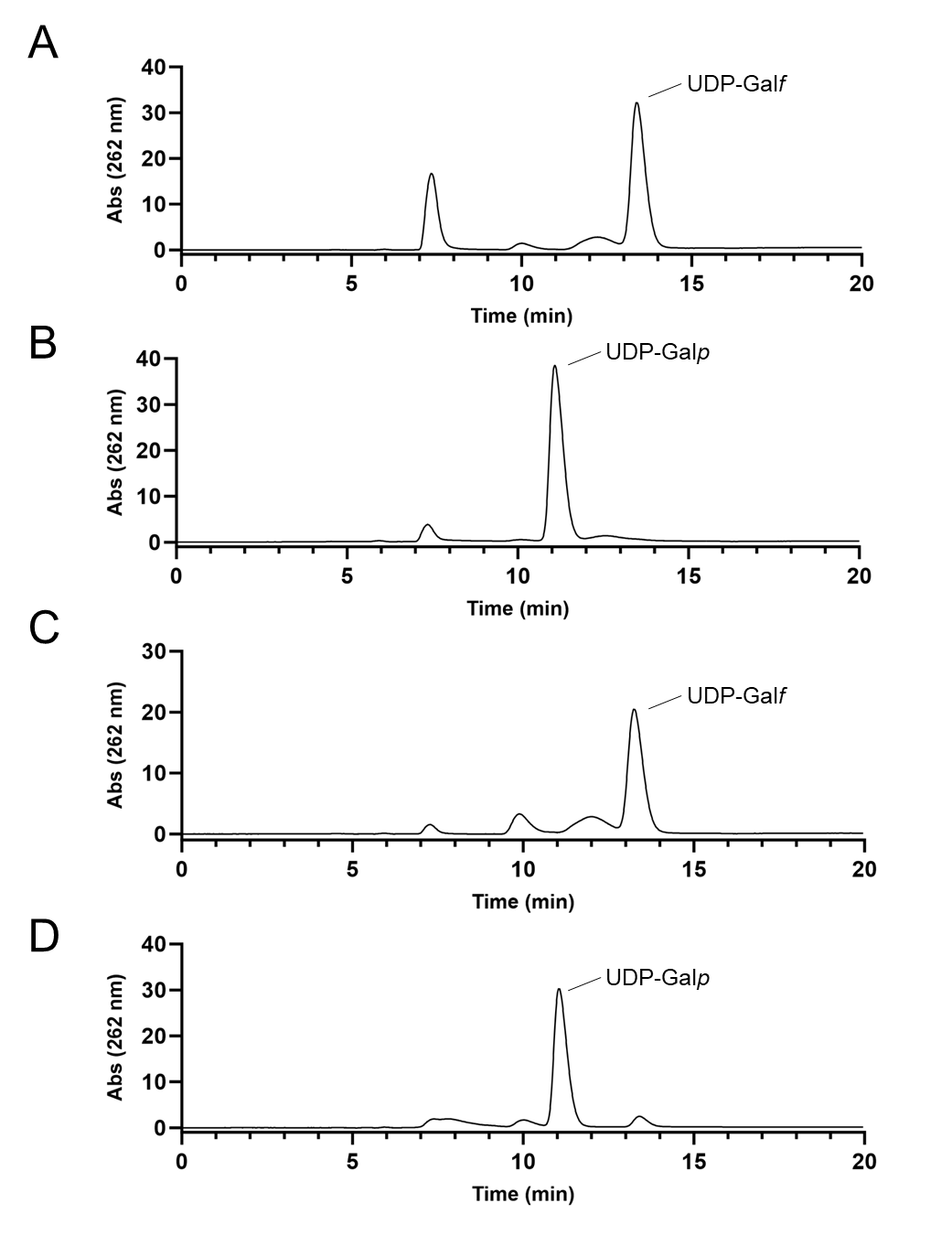


UDP-Gal*f*

*+* rPamB

UDP-Gal*f*

*+* rPamC

UDP-Gal*p*

UDP-Gal*f*

**Figure S2. PamB UDP-galactopyranose mutase assay.** (*A*, *B*) UDP-galactofuranase (UDP-Gal*f*) (*A*) and UDP-galactopyranose (UDP-Gal*p*) (*B*) controls were subject to HPLC resulting in peaks at 13.5 min and 11.1 min, respectively. The secondary peak at 7.3 min is likely free UDP. (*C*, *D*) UDP-Gal*f* was incubated with recombinant PamC1 (rPamC1) as a negative control condition (*C*) or with recombinant PamB (rPamB) (*D*), the predicted mutase, as previous studies on similar proteins have found that the reaction equilibrium significantly favors the UDP-Gal*f* to UDP-Gal*p* conversion (4, 5). The decrease in the 13.5 min peak and appearance of the 11.1 min peak in the PamB condition demonstrates that this protein is a UDP-Gal*p* mutase, capable of the interconversion of UDP-Gal*f* and UDP-Gal*p.*

**Figure S3. Pairwise amino acid alignment of PamC1 and PamC2.** EMBOSS Needle was used to construct a pairwise sequence alignment of PamC1 from strain KK01 (accession WP_003786867.1) and PamC2 from strain PYKK181 (accession AEK99054.1).

**Figure S4.** Synthetic Gal*f* acceptors.

**Figure S5. Clustal Omega multiple sequence alignment of GlfT2, PamC1, and PamC2.** Clustal Omega v1.2.4 was used to align the *M. tuberculosis* GlfT2 (accession NP_218325) and *K. kingae* PamC1 (accession WP_003786867.1) and PamC2 (accession AEK99054.1) proteins. The conserved DXD and DD critical sites targeted for mutational studies are in red boxes.


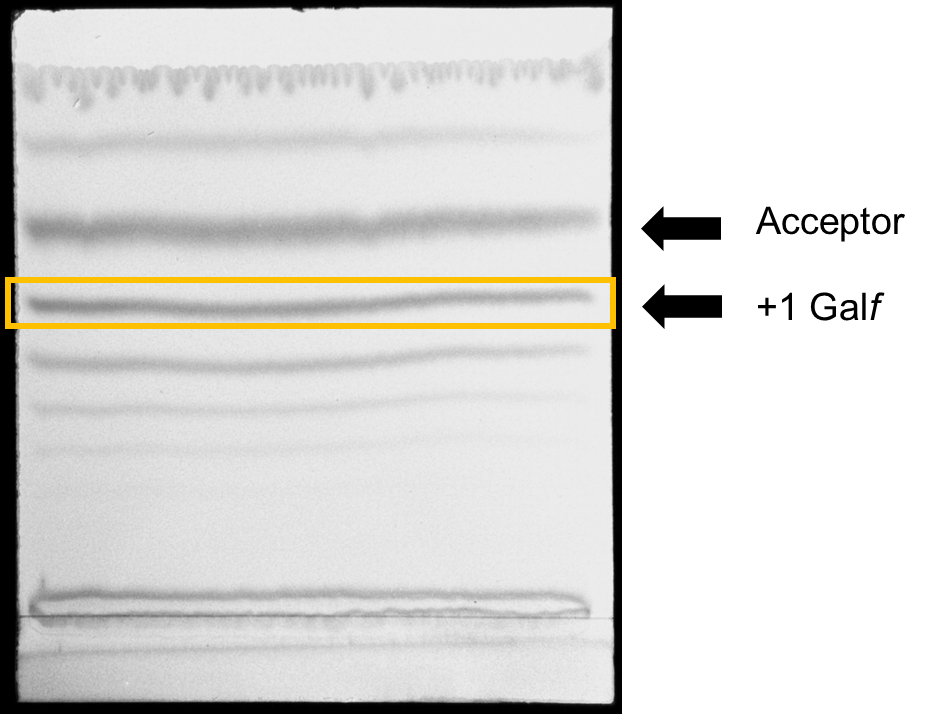


**Figure S6. Preparative thin layer chromatography (prep TLC).** A representative preparative TLC foil is shown. The reaction products of rPamC1- or rPamC2-treated synthetic Gal*f* acceptors were separated, and the silica containing the +1 Gal*f* reaction product (shown in the yellow box) was scraped from the TLC foil. Following extraction from the silica and purification, the product was subjected to ^1^H-NMR analysis.

|  |  | [ppm] | | | | | |
| --- | --- | --- | --- | --- | --- | --- | --- |
| **Glycosyl residue** |  | **H1** | **H2** | **H3** | **H4** | **H5** | **H6** |
|  |  |  |  |  |  |  |  |
| →5)-β-D-Gal*f*-(1→ |  | 5.18 | 4.14 | 3.94 | 4.15 | 4.11 | 3.80/3.80 |
|  |  |  |  |  |  |  |  |

**Table S1.** Proton chemical shift assignments of the *K. kingae* PYKK58 galactan. The observed signals are consistent with the [🡪5)-β-D-Gal*f*-(1🡪]_n_ galactan found in strain KK01 (6).

**Table S2.** Proton chemical shift assignments of the *K. kingae* PYKK143 galactan. The observed signals are consistent with the [🡪3)-β-Gal*f*-(1🡪6)-β-Gal*f*-(1🡪]_n_ galactan found in strain PYKK181 (7).

| . |  | [ppm] | | | | | |
| --- | --- | --- | --- | --- | --- | --- | --- |
| **Glycosyl residue** |  | **H1** | **H2** | **H3** | **H4** | **H5** | **H6** |
|  |  |  |  |  |  |  |  |
| →6)-β-D-Gal*f*-(1→ | **A** | 5.17 | 4.12 | 4.08 | 3.95 | 3.98 | 3.88/3.65 |
|  |  |  |  |  |  |  |  |
| →3)-β-D-Gal*f*-(1→ | **B** | 5.07 | 4.30 | 4.16 | 4.09 | 3.91 | 3.65/3.71 |

**Table S3. Bacterial strains and plasmids used in this study.**

| Strain or Plasmid | Description | Source |
| --- | --- | --- |
| *K. kingae* |  |  |
| KK01 | Nonspreading/noncorroding derivative of clinical isolate 269–492 | (8) |
| KK01*csaA* | KK01 with unmarked deletion of the capsule synthesis gene *csaA.* All KK01 background strains used in this study contain the *csaA* deletion. | (9) |
| KK01*pamABC* | KK01*csaA* with a kanamycin-marked *pamABC* deletion | (9) |
| KK01*pamDE* | KK01*csaA* with a kanamycin-marked *pamDE* deletion | (9) |
| KK01*pamA* | KK01*csaA* with *pamA* nonsense mutations | This study |
| KK01*pamB* | KK01*csaA* with *pamB* nonsense mutations | This study |
| KK01*pamC* | KK01*csaA* with *pamC* nonsense mutations | This study |
| KK01*pamA-*C*pamA* | KK01*pamA* with *pamA* complemented at an unlinked locus under control of the *pamA* promoter | This study |
| KK01*pamB-*C*pamB* | KK01*pamB* with *pamB* complemented at an unlinked locus under control of the *pilA1* promoter | This study |
| KK01*pamC-*C*pamC* | KK01*pamC* with *pamC* complemented at an unlinked locus under control of the *pilA1* promoter | This study |
| KK01+PamC2 | KK01*csaA* with its native *pamC1* allele replaced with *pamC2* from PYKK181 | This study |
| KK01+PamC1_D239A/D241A_ | KK01*csaA* encoding mutant PamC1_D239A/D241A_ | This study |
| KK01+PamC1_D343S_ | KK01*csaA* encoding mutant PamC1_D343S_ | This study |
| KK01+PamC2_D239A/D241A_ | KK01*csaA* encoding mutant PamC2_D239A/D241A_ | This study |
| KK01+PamC2_D343S_ | KK01*csaA* encoding mutant PamC2_D_**_343S_** | This study |
| PYKK58*csb* | *pamC1-*encoding strain PYKK58 with deletion of the *csb* capsule synthesis locus | (10) |
| PYKK181 | prototype *pamC2-*encoding strain | (7) |
| PYKK143 | Readily transformable *pamC2-*encoding strain | (10) |
| PYKK143*csb* | PYKK143 with a deletion of the *csb* capsule synthesis locus | This study |
| PYKK143+PamC2 | PYKK143*csb* with its native *pamC2* allele replaced with *pamC1* from KK01 | This study |
| *E. coli* |  |  |
| JM109 | *end*A1, *rec*A1, *gyr*A96, *thi, hsd*R17 (r_k_^–^,m_k_^+^), *rel*A1, *sup*E44, Δ( *lac-pro*AB), [F´ *tra*D36, *pro*AB, *laq*I^q^ZΔM15]. | Promega |
| DH5α | ﻿$\lambda$^—^$\phi$80d*lac*ZΔM15 Δ(*lacZYA-argF*)*U169* *recA1 endA1* hsdR17(r_K_^—^ m_K_^—^) *supE44 thi-1* *gyrA relA1* | Thermo Fisher Scientific |
| BL21(DE3) | *E. coli B F^-^ dcm ompT hsdS(rB-mB-) gal λ (DE3)* | Thermo Fisher Scientific |
| Plasmids |  |  |
| pUC19 | High copy number cloning vector | This study |
| pFalcon2 | Source of the *aphA3* kanamycin resistance cassette |  |
| pKan-*pamABC* | pUC19 containing the *pamABC* locus with an *aphA3* cassette upstream of *pamA* | This study |
| pKan-*pamA*stop | pKan-*pamABC* with *pamA* containing nonsense mutations | This study |
| pKan-*pamB*stop | pKan-*pamABC* with *pamB* containing nonsense mutations | This study |
| pKan-*pamA*stop | pKan-*pamABC* with *pamC* containing nonsense mutations | This study |
| pComp-Erm | Complementation plasmid for introducing *ermC*-marked (erythromycin)chromosomal complements into *K. kingae* | (11) |
| pComp*_pilA1_* | pComp-Erm containing the *K. kingae* *pilA1* promoter to drive expression of a complemented gene. | This study |
| pComp-*pamA* | pComp-Erm containing *pamA* for complementation | This study |
| pComp*_pilA1_*-*pamB* | pComp*_pilA1_* containing *pamB* for complementation | This study |
| pComp*_pilA1_*-*pamC* | pComp*_pilA1_* containing *pamC* for complementation | This study |
| pTrc99a | IPTG-inducible expression plasmid | (12) |
| pTrc99a-*pamABC* | pTrc99a containing IPTG-inducible *pamABC* | (9) |
| pTrc-*pamABC∆pamA* | pTrc99a-*pamABC* containing nonsense mutations in *pamA* | This study |
| pTrc-*pamABC∆pamB* | pTrc99a-*pamABC* containing nonsense mutations in *pamB* | This study |
| pTrc-*pamABC∆pamC* | pTrc99a-*pamABC* containing nonsense mutations in *pamC* | This study |
| p01*pamCD* | pUC19 containing SalI/PstI-flanked *pamCD* from KK01 | This study |
| p01Swap | p01pamCD containing the KK01 *pamAB* locus with an *aphA3* cassette upstream of *pamA* | This study |
| p181Swap | Gibson-assembled construct the KK01 *pamAB* locus with an *aphA3* cassette upstream and *pamCD* from PYKK181 | This study |
| p01Swap_D239A/D241A_ | p01Swap mutagenized to encode PamC1_D239A/D241A_ | This study |
| p01Swap_D343S_ | p01Swap mutagenized to encode PamC1_D343S_ | This study |
| p181Swap_D239A/D241A_ | p181Swap mutagenized to encode PamC2_D239A/D241A_ | This study |
| p181Swap_D343S_ | p181Swap mutagenized to encode PamC2_D343S_ | This study |
| pHAT10 | *E. coli* Histidine Affinity Tag (HAT) N-terminal fusion recombinant protein expression plasmid | Takara Bio |
| pHAT-PamA | pHAT10 encoding the HAT-PamA fusion (from KK01) | This study |
| pHAT-PamB | pHAT10 encoding the HAT-PamB fusion (from KK01) | This study |
| pHAT-PamC1 | pHAT10 encoding the HAT-PamC1 fusion (from KK01) | This study |
| pHAT-PamC2 | pHAT10 encoding the HAT-PamC2 fusion (from PYKK181) | This study |
| pHAT-PamC1_D239A/D241A_ | pHAT10 encoding the HAT-PamC1_D239A/D241A_ fusion (from KK01) | This study |
| pHAT-PamC1_D343S_ | pHAT10 encoding the HAT-PamC1_D343S_ fusion (from KK01) | This study |
| pHAT-PamC2_D239A/D241A_ | pHAT10 encoding the HAT-PamC2_D239A/D241A_ fusion (from PYKK181) | This study |
| pHAT-PamC2_D343S_ | pHAT10 encoding the HAT-PamC2_D343S_ fusion (from PYKK181) | This study |

**Table S4. Primers used in this study.**

| Primer name | Sequence (5’→3’) |
| --- | --- |
| pamAupF | ACGTGAATTCTTGGCGAGCGTGGTTTATC |
| pamAupR | ACGTGGTACCAAATTGCAGGCTGCTTTTCAC |
| pamABC_markedF | ACGTGGATCCGCCTGCAATTTGATAGAATAATAAAAC |
| pamABC_markedR | ACGTGTCGACACCGTTGGCAAACCTTCG |
| aphA3_KpnIF | GCATGGTACCGTTTGACAGCTTATCATCGATAAACCCAG |
| aphA3_BamHIR | GCATGGATCCCATCTAAATCTAGGTACTAAAACAATTCATCCAG |
| pamA mut F | CGCGTAAATAGCCTAGATTTTATGGCTAATTACCTAATTGCAATGGCAC |
| pamA mut R | GTGCCATTGCAATTAGGTAATTAGCCATAAAATCTAGGCTATTTACGCG |
| pamB mut F | GCGGTTGGTATAAGGCATAAATTAGCCAAATTACTGCAAATAATTCCATACAGTTT |
| pamB mut R | AAACTGTATGGAATTATTTGCAGTAATTTGGCTAATTTATGCCTTATACCAACCGC |
| pamC mut F | CGCAACCAGCTCCTTATTATAGGAAACGGATTTCTCTTTCATTTCG |
| pamC mut R | CGAAATGAAAGAGAAATCCGTTTCCTATAATAAGGAGCTGGTTGCG |
| pamA comp F | AGCTggatccGAATGCAGTGGCGGATATTG |
| pamA comp R | AGCTggtaccCCTAGTTATGTTTGTACCCGC |
| pamB comp F | ACGTtctagaATGAATTTTTTATGTGTCGGAGCAG |
| pamB comp R | AGCTGGTACCCTCAGACAGCCTTTTGCAG |
| pamC comp F | ACGTGGATCCATGAATATTATCCAAAATCTCGTTTTCC |
| pamC comp R | ACGTGGTACCCTATGAAAGGCTACCTGCAAAC |
| pam_unmark F | AACAATTTAAACAATTACCTGAGTCG |
| pam_unmark R | GAGGTTTGTTGCTTCGGAAC |
| PamA_HAT_F | ACGTGGATCCTTCCAATTAAGCGAAATTCCAAC |
| PamA_HAT_R | AGCTgaattcCCTAGTTATGTTTGTACCCGC |
| PamB_HAT_F | ACGTGGATCCAATTTTTTATGTGTCGGAGCAGG |
| PamB_HAT_R | AGCTGAATTCAAAATTAAGAAAAATAAAACGGTTTAATTTCC |
| PamC_HAT_F | ACGTGGATCCAATATTATCCAAAATCTCGTTTTCCC |
| PamC_HAT_R | AGCTGAATTCAAATTATTTTTCAGTGTTATAAATTTTTTCCCAG |
| pamC1Swap_F | ACGTGTCGACAGGCTGTCTGAGATATTTATCATTTC |
| pamC1Swap_R | ACGTCTGCAGGCTGGCTAGGGAGATGATATAGG |
| pamB_R1 | ACGTGTCGACTTTGCAGGCTGCCTGAAATG |
| pamC2Swap_F | atttcaggcagcctgcaaagtcgacaggctgtctgagatatttatcatttc |
| pamC2Swap_R | gccaagcttgcatgcctgcagctggctagggagatgatatag |
| pamC_DDD_sense | caatttcacaagacgcagcatcggccataaataagcagtgcgtata |
| pamC_DDD_anti | tatacgcactgcttatttatggccgatgctgcgtcttgtgaaattg |
| pamC1_D343S_sense | attaaatttatttgataaactaaaacaaatagaatcaccgcgtacaaaaaatgggaatgcatac |
| pamC1_D343S_anti | gtatgcattcccattttttgtacgcggtgattctatttgttttagtttatcaaataaatttaat |
| pamC2_D343S_sense | gttcataaagccgaatgaaacagaatcaccgcgaacaaagaaaggaaatg |
| pamC2_D343S_anti | catttcctttctttgttcgcggtgattctgtttcattcggctttatgaac |

**SUPPORTING METHODS**

**Synthesis of acceptors (compounds 1, 2, 3 and 4) for Gal*f*-PS polymerization**

*General Methods*

All chemicals and reagents were purchased from commercial sources and were used without further purification unless noted. Reaction solvents (THF and CH_2_Cl_2_) were taken from a solvent purification system in which the solvents were purified by successive passage through columns of alumina and copper under argon. Unless stated otherwise, all reactions were carried out at room temperature (rt) and under a positive pressure of argon and were monitored by TLC on Silica Gel G-25 F254 (0.25 mm, Merck). Visualization of the reaction components on TLC was achieved using UV light (254 nm) and/or by charring after treatment with a solution of *p*-anisaldehyde (3.7 mL) and glacial acetic acid (1.5 mL) and concentrated H_2_SO_4_ (5 mL) in ethanol (135 mL). Organic solvents were evaporated under reduced pressure, and the products were purified by column chromatography on silica gel (70 mesh). Optical rotations were measured on a Jasco P-2000 digital polarimeter at the sodium D line (589 nm) at 25 ± 2 °C and are in units of (deg·mL)/(dm·g). NMR spectra were recorded on BRUKER AVANCE 500 and BRUKER AVANCE III 600 spectrometers. ^1^H NMR spectra were recorded at 500 MHz or 600 MHz, and the chemical shifts are referenced to residual CHCl_3_ (7.26 ppm, CDCl_3_) or HDO (4.78 ppm, D_2_O). ^13^C NMR spectra were recorded at 126 MHz or 151 MHz and are proton decoupled, and the chemical shifts are referenced to CDCl_3_ (77.0 ppm, CDCl_3_) or internal acetone (31.45 ppm, D_2_O). Standard splitting patterns are abbreviated: s (singlet), d (doublet), t (triplet), q (quartet), m (multiplet). To unequivocally assign the ^1^H and ^13^C NMR data, the protons and carbons corresponding to the monosaccharide at the reducing end were unprimed, while those corresponding to the next monosaccharide were labelled as H' and C', and the next furthest from the reducing end H'' and C'', and so on. High resolution and high mass accuracy LC-MS experiments were done on a LTQFT Ultra (Linear quadrupole ion trap Fourier transform ion cyclotron resonance) mass spectrometer (Thermo Electron, San Jose, CA) equipped with a HESI-II source, an Agilent 1100 Series binary high-performance liquid chromatography pump (Agilent Technologies, Palo Alto, CA), and a Famos autosampler (LC Packings, San Francisco, CA). High resolution MALDI–TOF mass spectra were conducted on a New ultrafleXtreme^TM^ MALDI–TOF/TOF mass spectrometer (Bruker Corporation, Bremen, Germany) using DHB (2,5-dihydroxybezoic acid) as the matrix.

*Synthesis of acceptors* ***1–4.***

**8-(4-methoxybenzamido)-1-octyl β-D-galactofuranosyl-(1→5)-β-D-galactofuranoside (1).** Triphenylphosphine (21.0 mg, 0.0808 mmol) and 6N NaOH (2.0 µL) was added to a solution of **10** (20.0 mg, 0.0404 mmol) in THF–H_2_O (5 mL, 4:1). The solution was stirred overnight before the solvent was evaporated. The resulting crude mixture was dissolved in CH_3_OH (4 mL) followed by the addition of 4-methoxybenzoyl chloride (8.2 µL, 0.0606 mmol) and triethylamine (16.4 µL, 0.121 mmol). After 3 h of stirring, the mixture was concentrated to dryness. The crude residue was purified by reversed phase column chromatography (0% to 40% gradient of CH_3_OH in H_2_O) using a C18 Sep-Pak cartridge column to give a product that was redissolved in distilled water. The resulting solution was frozen and then lyophilized to afford **1** (20.2 mg, 83%) as a white solid: [α]_D_^25^ –71.0 (*c* = 0.65, H_2_O); ^1^H NMR (600 MHz, D_2_O) δ 7.75–7.70 (m, 2H, Ar), 7.04–6.99 (m, 2H, Ar), 5.21 (d, *J* = 1.9 Hz, 1H, H-1$’$), 4.93 (d, *J* = 2.4 Hz, 1H, H-1), 4.15 (dd, *J* = 3.8, 2.0 Hz, 1H, H-2’), 4.10 (dd, *J* = 7.0, 4.3 Hz, 1H, H-3), 4.10–4.04 (m, 2H, H-3’, H-4’), 4.04–3.99 (m, 2H, H-4, H-2), 3.93 (app td, *J* = 5.8, 3.2 Hz, 1H, H-5), 3.84 (s, 3H, OCH_3_), 3.87–3.81 (m, 1H, H-5’), 3.80–3.74 (m, 2H, H-6a, H-6b), 3.72 (dd, *J* = 11.7, 4.6 Hz, 1H, H-6a’), 3.70–3.63 (m, 2H, H-6b’, octyl OCH_2_), 3.50 (app dt, *J* = 9.9, 6.5 Hz, 1H, octyl OCH_2_), 3.34 (app t, *J* = 6.9 Hz, 2H, octyl CH_2_NH), 1.36–1.25 (m, 8H, 4 x CH_2_); ^13^C NMR (151 MHz, D_2_O) δ 169.8 (C=O), 161.8 (Ar), 128.9 (2 x Ar), 126.3 (Ar), 113.9 (2 x Ar), 107.2 (C-1’), 107.0 (C-1), 82.6 (C-1’), 81.3 (C-4’), 81.2 (C-2’), 81.1 (C-2), 76.5 (C-3’), 76.3 (C-3), 75.8 (C-5), 70.5 (C-5’), 68.5 (octyl O*C*H_2_), 62.8 (C-6’), 61.2 (C-6), 55.4 (OCH_3_), 39.9 (octyl CH_2_NH), 28.6 (CH_2_), 28.4 (CH_2_), 28.3 (CH_2_), 28.3 (CH_2_), 26.1 (CH_2_), 25.1 (CH_2_); HRMS (ESI–TOF) *m/z* [M + H]^+^ calcd for C_28_H_46_NO_13_^+^ 604.2964, found 604.2966.

The synthesis of compounds **2** and **3** were previously described (13).

**8-(4-methoxybenzamido)-1-octyl β-D-galactofuranoside (4).** Triphenylphosphine (47.0 mg, 0.180 mmol) and 6N NaOH (2.0 µL) were added to a solution of **13** (30.0 mg, 0.0900 mmol) in THF–H_2_O (5 mL, 4:1). The solution was stirred overnight before the solvent was evaporated. The resulting crude mixture was dissolved in CH_3_OH (5 mL) followed by the addition of 4-methoxybenzoyl chloride (18.0 µL, 0.135 mmol) and triethylamine (36.0 µL, 0.270 mmol). After 3 h of stirring, the mixture was concentrated to dryness. The crude residue was purified by reversed phase column chromatography (0% to 40% gradient of CH_3_OH in H_2_O) using a C18 Sep-Pak cartridge column to give a product that was redissolved in distilled water. The resulting solution was frozen and then lyophilized to afford **4** (37.0 mg, 93%) as a white solid: [α]_D_^25^ –70.0 (*c* = 0.09, CH_3_OH); ^1^H NMR (600 MHz, MeOD) δ 7.81–7.75 (m, 2H, Ar), 7.00–6.94 (m, 2H, Ar), 4.84 (d, *J* = 2.0 Hz, 1H, H-1), 4.00 (dd, *J* = 6.7, 4.0 Hz, 1H, H-3), 3.94 (dd, *J* = 4.0, 2.0 Hz, 1H, H-2), 3.91 (dd, *J* = 6.7, 3.2 Hz, 1H, H-4), 3.84 (s, 3H, OCH_3_), 3.73–3.67 (m, 2H, H-5, octyl OCH_2_), 3.65–3.59 (m, 2H, H-6a, H-6b), 3.41 (app dt, *J* = 9.7, 6.6 Hz, 1H, octyl OCH_2_), 3.35 (app t, *J* = 7.2 Hz, 2H, octyl CH_2_NH), 1.65–1.55 (m, 4H, OCH_2_C*H*_2,_ C*H*_2_CH_2_NH), 1.41–1.34 (m, 8H, 4 x CH_2_); ^13^C NMR (151 MHz, MeOD) δ 168.4 (C=O), 162.4 (Ar), 128.7 (2 x Ar), 126.5 (Ar), 113.3 (2 x Ar), 108.0 (C-1), 82.7 (C-4), 82.1 (C-2), 77.3 (C-3), 71.0 (C-5), 67.5 (octyl O*C*H_2_), 63.2 (C-6), 54.5 (OCH_3_), 39.6 (octyl CH_2_NH), 29.3 (CH_2_), 29.2 (CH_2_), 29.0 (2 x CH_2_), 26.7 (CH_2_), 25.8 (CH_2_); HRMS (ESI–TOF) *m/z* [M + H]^+^ calcd for C_22_H_36_N_4_O_8_^+^ 442.2435, found 442.2438.

*Synthesis of disaccharide* ***10***

**2,3,5-Tri-*O*-benzoyl-6-*O*-levulinoyl-β-D-galactofuranosyl fluoride (6).** *N*,*N*-diethylaminosulfur trifluoride (26.0 µL, 0.177 mmol) and *N*-bromosuccinimide (21.1 mg, 0.118 mmol) were added at 0 °C under Ar atmosphere to a solution of **5** (15) (68.8 mg, 98.7 µmol) in CH_2_Cl_2_ (3.0 mL). The reaction mixture was stirred for 1 h at 0 °C, then MeOH (1 mL) was added. The mixture was diluted with EtOAc and washed with sat. aq. NaHCO_3_ and brine. The organic layer was dried over Na_2_SO_4_, filtered and concentrated. The residue was purified by silica gel flash column chromatography (2:1 *n*-hexane–EtOAc) to give compound **6** (57.2 mg, 98%): *R_f_* 0.58 (1:1 *n*-hexane–EtOAc); [α]_D_^25^ +8.6 (*c* 1.0, CH_2_Cl_2_); H^1^ NMR (500 MHz, CDCl_3_) δ 8.08–8.04 (m, 4H, Ar), 7.88–7.86 (m, 2H, Ar), 7.62–7.58 (m, 1H, Ar), 7.55–7.51 (m, 2H, Ar), 7.48–7.45 (m, 2H, Ar), 7.35–7.32 (m, 2H, Ar), 7.30–7.27 (m, 2H, Ar), 6.03 (d, 1H, *J* = 58.1 Hz, H-1), 5.93 (app td, 1H, *J* = 7.0, 4.4 Hz, H-5), 5.64 (dd, 1H, *J* = 6.5, 0.8 Hz, H-2), 5.60 (d, 1H, *J* = 4.3 Hz, H-3), 4.81(app td, 1H, *J* = 4.1, 1.4 Hz, H-4), 4.56 (dd, 1H, *J* = 11.9, 4.5 Hz, H-6a), 4.48 (dd, 1H, *J* = 11.8, 7.2 Hz, H-6b), 2.68–2.65 (m, 2H, CH_2_C*H_2_*COO), 2.55–2.52 (m, 2H, CH_2_C*H_2_*CO), 2.09 (s, 3H, CH_3_CO); C^13^ NMR (125 MHz, CDCl_3_) δ 206.3, 172. 3, 165.8, 165.7, 165.3, 133.9, 133.8, 133.5, 130.1, 129.6, 129.4, 128.8, 128.7, 128.61, 128.58, 128.5, 112.5 (d, *J* = 226.4 Hz, C-1), 84.8 (C-4), 81.1 (d, *J* = 40.2 Hz, C-2), 76.6 (C-3), 70.1 (C-5), 63.0 (C-6), 37.9 (CH_2_*C*H_2_COO), 29.8 (*C*H_3_CO), 27.9 (CH_2_*C*H_2_CO); HRMS (ESI–TOF) *m/z* [M + Na]^+^ calcd for C_32_H_29_FNaO_10_^+^ 615.1637, found 615.1630.

**4-Methylphenyl 2,3,5-tri-*O*-benzoyl-6-*O*-levulinoyl-β-D-galactofuranosyl-(1→5)-2,3,6-tri-*O*-benzoyl-1-thio-β-D-galactofuranoside (8).** A mixture of **6** (55.8 mg, 94.2 µmol), **7** (47.0 mg, 78.5 µmol) and 4Å MS (200 mg) in toluene (3.00 mL) was stirred under an Ar atmosphere for 1 h at room temperature. The mixture was cooled to 0 °C, then Cp_2_ZrCl_2_ (33.0 mg, 0.113 mmol) and AgOTf (58.1 mg, 0.226 mmol) were added. After the reaction mixture was stirred for 2 h at 0 °C, additional Cp_2_ZrCl_2_ (8.3 mg, 28.3 µmol) and AgOTf (14.5 mg, 56.5 µmol) were added. After stirring for another 1 h at 0 °C, triethylamine was added to the reaction mixture, then the mixture was filtered through Celite and washed with satd aq NaHCO_3_ and brine. The organic layer was dried over Na_2_SO_4_, filtered and concentrated. The residue was purified by column chromatography (10:1 toluene–EtOAc) to give **8** (84.4 mg, 92%) as a white foam: *R_f_*= 0.63 (4:1 toluene–acetone); [α]_D_^25^ –60.3 (*c* = 1.0, CHCl_3_); ^1^H NMR (500 MHz, CDCl_3_) δ 8.06–8.00 (m, 6H, Ar), 7.97–7.95 (m, 2H, Ar),7.87–7.85 (m, 2H, Ar), 7.83–7.81 (m, 2H, Ar), 7.58–7.56 (m, 2H, Ar), 7.53–7.42 (m, 9H, Ar), 7.38–7.34 (m, 2H, Ar), 7.32–7.21 (m, 8H, Ar), 7.06 (d, 2H, *J* = 8.5 Hz, Ar), 5.90 (dd, 1H, *J* = 5.1, 0.7 Hz, H-3’), 5.84 (app td, 1H, *J* = 7.7, 3.9 Hz, H-5’), 5.75 (s, 1H, H-1’), 5.72 (d, 1H, *J* = 1.7 Hz, H-1), 5.69 (app t, 1H, *J* = 2.2 Hz, H-2), 5.63 (d, 1H, *J* = 1.2 Hz, H-2’), 5.56 (dd, 1H, *J* = 5.2, 0.9 Hz, H-3), 4.88 (dd, 1H, *J* = 5.2, 3.5 Hz, H-4’), 4.80 (dd, 1H, *J* = 4.9, 3.2 Hz, H-4), 4.76–4.68 (m, 3H, H-5, H-6a, H-6b), 4.46 (dd, 1H, *J* = 11.8, 4.3 Hz, H-6’a), 4.38 (dd, 1H, *J* = 7.7, 11.8 Hz, H-6’b), 2.58–2.53 (m, 2H, CH_2_C*H_2_*COO), 2.46–2.43 (m, 2H, CH_2_C*H_2_*CO), 2.29 (s, 3H, PhCH_3_), 2.02 (s, 3H, CH_3_CO); ^13^C NMR (125 MHz, CDCl_3_) δ 206.3, 172.3, 166.2, 165.8, 165.7, 165.6, 165.4, 165.3, 138.2, 133.7, 133.6, 133.4, 133.3, 133.1, 132.8, 130.11, 130.06, 130.0, 129.94, 129.91, 129.89, 129.8, 129.61, 129.56, 129.1, 129.00, 128.95, 128.92, 128.86, 128.67, 128.65, 128.5, 128.4, 128.3, 105.6 (C-1’), 91.3 (C-1), 82.4 (C-4), 82.3 (C-4’), 82.1 (C-2), 81.6 (C-2’), 77.8 (C-3), 77.4 (C-3’), 73.6 (C-5), 70.4 (C-5’), 64.5 (C-6), 63.3 (C-6’), 37.9 (CH_2_*C*H_2_COO), 29.7 (*C*H_3_CO), 27.9 (CH_2_*C*H_2_CO), 21.2 (PhCH_3_); HRMS (ESI–TOF) *m/z* [M + Na]^+^ calcd for C_66_H_58_NaO_18_S^+^ 1193.3236, found 1193.3228.

**8-azido-1-octyl 2,3,5-tri-*O*-benzoyl-6-*O*-levulinoyl-β-D-galactofuranosyl-(1→5)-2,3,6-tri-*O*-benzoyl-β-D-galactofuranoside (9).** To a stirred solution of donor **8** (100 mg, 0.0853 mmol) and 8-azido-1-octanol (22.0 mg, 0.128 mmol) in dry CH_2_Cl_2_ (5 mL) was added 4Å molecular sieves powder (300 mg). After stirring for 30 min at room temperature, the reaction mixture was cooled to 0 °C, and then *N*-iodosuccinimide (29.0 mg, 0.128 mmol) and silver trifluoromethanesulfonate (4.4 mg, 0.017 mmol) were added successively. The resulting solution was warmed to room temperature and stirred for 2 h. Triethylamine was added to the mixture and the solution was diluted with CH_2_Cl_2_ and then filtered through Celite. The filtrate was washed with saturated aqueous Na_2_S_2_O_3_ and saturated aqueous NaHCO_3_. The organic layer was dried over Na_2_SO_4_, filtered and concentrated to dryness. The crude residue was purified by flash chromatography (2:1 hexanes–EtOAc) to afford **9** (88 mg, 85%) as a white solid: *R_f_* = 0.16 (2:1 hexanes–EtOAc); [α]_D_^25^ –10.5 (*c* = 1.25, CHCl_3_); ^1^H NMR (500 MHz, CDCl_3_) δ 8.04–7.97 (m, 8H, Ar), 7.85–7.79 (m, 4H, Ar), 7.58–7.53 (m, 1H, Ar), 7.52–7.46 (m, 4H, Ar), 7.45–7.38 (m, 3H, Ar), 7.38–7.34 (m, 3H, Ar), 7.34–7.29 (m, 2H, Ar), 7.29–7.24 (m, 4H, Ar), 7.24–7.20 (m, 2H, Ar), 5.87 (app dt, *J* = 7.6, 3.7 Hz, 1H, H-5’), 5.80 (dd, *J* = 5.1, 1.5 Hz, 1H, H-3), 5.77 (s, 1H, H-1’), 5.64 (d, *J* = 1.5 Hz, 1H, H-2’), 5.53 (dd, *J* = 5.2, 1.4 Hz, 1H, H-3’), 5.49 (d, *J* = 1.5 Hz, 1H, H-2), 5.21 (s, 1H, H-1), 4.91 (dd, *J* = 5.2, 3.4 Hz, 1H, H-4’), 4.77 (app dt, *J* = 9.3, 6.9 Hz, 1H, H-6a), 4.72–4.65 (m, 2H, H-6b, H-5), 4.55 (dd, *J* = 11.9, 3.9 Hz, 1H, H-6a’), 4.51 (dd, *J* = 5.2, 3.3 Hz, 1H, H-4), 4.40 (dd, *J* = 11.9, 7.9 Hz, 1H, H-6b’), 3.70 (app dt, *J* = 9.6, 6.7 Hz, 1H, octyl OCH_2_), 3.48 (app dt, *J* = 9.7, 6.3 Hz, 1H, octyl OCH_2_), 3.22 (app t, *J* = 7.0 Hz, 2H, octyl CH_2_N_3_), 2.65–2.51 (m, 2H, COCH_2_C*H*_2_COCH_3_), 2.50–2.44 (m, 2H, COC*H_2_*CH_2_COCH_3_), 2.04 (s, 3H, COCH_3_), 1.66–1.51 (m, 4H, OCH_2_C*H*_2_, C*H*_2_CH_2_N_3_), 1.41–1.22 (m, 8H, 4 x CH_2_); ^13^C NMR (151 MHz, CDCl_3_) δ 206.3 (C=O), 172.2 (C=O), 166.2 (C=O), 165.7 (C=O), 165.7 (C=O), 165.5 (C=O), 165.5 (C=O), 165.2 (C=O), 133.5 (2 x Ar), 133.3 (Ar), 133.3 (Ar), 133.2 (Ar), 133.0 (Ar), 130.0 (2 x Ar), 129.9 (2 x Ar), 129.8 (2 x Ar), 129.8 (4 x Ar), 129.7 (3 x Ar), 129.5 (Ar), 129.1 (Ar), 129.0 (Ar), 128.9 (Ar), 128.8 (Ar), 128.6 (2 x Ar), 128.5 (2 x Ar), 128.4 (6 x Ar), 128.2 (2 x Ar), 105.5 (C-1), 105.2 (C-1’), 82.4 (C-4), 82.1 (C-4’), 81.9 (C-2’), 81.8 (C-2), 77.8 (C-3’), 77.1 (C-3), 73.0 (C-5), 70.3 (C-5’), 67.5 (octyl O*C*H_2_), 64.6 (C-6), 63.3 (C-6’), 51.4 (octyl *C*H_2_N_3_), 37.8 (COCH_2_*C*H_2_COCH_3_), 29.7 (COCH_3_), 29.4 (CH_2_), 29.3 (CH_2_), 29.1 (CH_2_), 28.8 (CH_2_), 27.8 (CO*C*H_2_CH_2_COCH_3_), 26.7 (CH_2_), 26.0 (CH_2_); HRMS (ESI–TOF) *m/z* [M + NH_4_]^+^ calcd for C_67_H_71_N_4_O_19_^+^ 1235.4707, found 1235.4718.

**8-azido-1-octyl β-D-galactofuranosyl-(1→5)-β-D-galactofuranoside (10).** To a stirred solution of **9** (80.0 mg, 0.0657 mmol) in CH_3_OH–CH_2_Cl_2_ (10 mL, 4:1) was added a solution of NaOCH_3_ (0.5 M in CH_3_OH) at room temperature until the of the solution pH = 12. After stirring overnight, the reaction mixture was neutralized by the addition of Amberlite® IR-120 (H^+^) cation exchange resin, filtered and concentrated to dryness. The crude product was purified by flash chromatography (10:1 CH_2_Cl_2_–CH_3_OH) to a white solid that was redissolved in distilled water. The resulting solution was frozen and then lyophilized to afford **10** (25.0 mg, 77%) as a white solid: *R_f_* = 0.14 (10:1 CH_2_Cl_2_–CH_3_OH); [α]_D_^25^ –182.7 (*c* = 0.09, H_2_O); ^1^H NMR (600 MHz, D_2_O) δ 5.22 (d, *J* = 1.9 Hz, 1H, H-1’), 4.97 (d, *J* = 2.3 Hz, 1H, H-1), 4.16 (dd, *J* = 3.8, 2.0 Hz, 1H, H-2’), 4.12 (dd, *J* = 7.0, 4.3 Hz, 1H, H-3), 4.10–4.06 (m, 2H, H-3’, H-4’), 4.06–4.05 (m, 1H, H-4), 4.04 (dd, *J* = 4.2, 2.4 Hz, 1H, H-2), 3.95 (app td, *J* = 5.8, 3.3 Hz, 1H, H-5), 3.85 (app dt, *J* = 7.6, 4.1 Hz, 1H, H-5’), 3.81–3.77 (m, 2H, H-6a, H-6b), 3.73 (app dt, *J* = 10.1, 6.7 Hz, 1H, octyl OCH_2_), 3.72 (dd, *J* = 11.8, 4.5 Hz, 1H, H-6a’), 3.68 (dd, *J* = 11.7, 7.3 Hz, 1H, H-6b’), 3.64–3.61 (m, 1H, H-6b), 3.58 (app dt, *J* = 10.1, 6.6 Hz, 1H, octyl OCH_2_), 3.33 (app t, *J* = 6.9 Hz, 2H, octyl CH_2_N_3_), 1.66–1.56 (m, 4H, OCH_2_C*H*_2,_ C*H*_2_CH_2_NH), 1.41–1.31 (m, 8H, 4 x CH_2_); ^13^C NMR (151 MHz, D_2_O) δ 107.1 (C-1’), 106.9 (C-1), 82.6 (C-4’), 81.3 (C-2’, C-2), 81.0 (C-4), 76.5 (C-3’), 76.3 (C-3), 75.9 (C-5), 70.5 (C-5’), 68.6 (octyl O*C*H_2_), 62.8 (C-6’), 61.1 (C-6), 51.2 (octyl CH_2_N_3_), 28.6 (CH_2_), 28.2 (CH_2_), 28.1 (CH_2_), 27.9 (CH_2_), 25.8 (CH_2_), 25.0 (CH_2_); HRMS (ESI–TOF) *m/z* [M + Na]^+^ calcd for C_20_H_37_N_3_NaO_11_^+^ 518.2320, found 518.2323.

*Synthesis of monosaccharide* ***13***

******

**8-azido-1-octyl 2,3,5,6-tetra-*O*-benzoyl-β-D-galactofuranoside (12).** 4Å molecular sieves powder (300 mg) was added to a stirred solution of donor **11** (14) (100 mg, 0.142 mmol) and 8-azido-1-octanol (36.0 mg, 0.213 mmol) in dry CH_2_Cl_2_ (5 mL). After stirring for 30 min at room temperature, the reaction mixture was cooled to 0 °C, and then *N*-iodosuccinimide (48.0 mg, 0.213 mmol) and silver trifluoromethanesulfonate (7.0 mg, 0.028 mmol) were added successively. The resulting solution was warmed to room temperature and stirred for 2 h. Triethylamine was added to the mixture, and the solution was diluted with CH_2_Cl_2_ and then filtered through Celite. The filtrate was washed with saturated aqueous Na_2_S_2_O_3_ and saturated aqueous NaHCO_3_. The organic layer was dried over Na_2_SO_4_, filtered and concentrated to dryness. The crude residue was purified by flash chromatography (4:1 hexanes–EtOAc) to afford **12** (100 mg, 94%) as a colorless oil: *R_f_* = 0.32 (4:1 hexanes–EtOAc); [α]_D_^25^ –13.1 (*c* = 0.11, CHCl_3_); ^1^H NMR (600 MHz, CDCl_3_) δ 8.10–8.06 (m, 2H, Ar), 8.06–8.03 (m, 2H, Ar), 8.00–7.95 (m, 2H, Ar), 7.93–7.88 (m, 2H, Ar), 7.59–7.54 (m, 1H, Ar), 7.55–7.49 (m, 2H, Ar), 7.45–7.40 (m, 2H, Ar), 7.40–7.35 (m, 2H, Ar), 7.33–7.27 (m, 4H, Ar), 6.07 (app dt, *J* = 7.6, 4.0 Hz, 1H, H-5), 5.63 (d, *J* = 5.2 Hz, 1H, H-3), 5.46 (d, *J* = 1.2 Hz, 1H, H-2), 5.30 (s, 1H, H-1), 4.78 (dd, *J* = 11.8, 4.4 Hz, 1H, H-6a), 4.74 (dd, *J* = 11.8, 7.2 Hz, 1H, H-6b), 4.64 (dd, *J* = 5.2, 3.5 Hz, 1H, H-4), 3.75 (app dt, *J* = 9.6, 6.8 Hz, 1H, octyl OCH_2_), 3.54 (app dt, *J* = 9.6, 6.3 Hz, 1H, octyl OCH_2_), 3.22 (app t, *J* = 7.0 Hz, 2H, octyl CH_2_N_3_), 1.70–1.62 (m, 2H, OCH_2_C*H*_2,_), 1.60–1.52 (m, 2H, C*H*_2_CH_2_N_3_), 1.44–1.23 (m, 8H, 4 x CH_2_); ^13^C NMR (151 MHz, CDCl_3_) δ 166.3 (C=O), 165.9 (C=O), 165.8 (C=O), 165.6 (C=O), 133.6 (Ar), 133.5 (Ar), 133.4 (Ar), 133.2 (Ar), 130.1 (2 x Ar), 130.1 (2 x Ar), 130.0 (2 x Ar), 129.9 (2 x Ar), 129.7 (Ar), 129.6 (Ar), 129.2 (Ar), 129.1 (Ar), 128.5 (6 x Ar), 128.5 (2 x Ar), 105.7 (C-1), 82.2 (C-2), 81.4 (C-4), 77.7 (C-3), 70.4 (C-5), 67.7 (octyl O*C*H_2_), 63.7 (C-6), 51.6 (octyl *C*H_2_N_3_), 29.6 (CH_2_), 29.4 (CH_2_), 29.2 (CH_2_), 28.9 (CH_2_), 26.8 (CH_2_), 26.2 (CH_2_); HRMS (ESI–TOF) *m/z* [M + NH_4_]^+^ calcd for C_42_H_47_N_4_O_10_^+^ 767.3287, found 767.3286.

**8-azido-1-octyl β-D-galactofuranoside (13).** To a stirred solution of **12** (80.0 mg, 0.107 mmol) in CH_3_OH–CH_2_Cl_2_ (10 mL, 4:1) was added a solution of NaOCH_3_ (0.5 M in CH_3_OH) at room temperature until the of the solution pH = 12. After stirring overnight, the reaction mixture was neutralized by the addition of Amberlite® IR-120 (H^+^) cation exchange resin, filtered and concentrated to dryness. The crude product was purified by flash chromatography (10:1 CH_2_Cl_2_–CH_3_OH) to a white solid that was redissolved in distilled water. The resulting solution was frozen and then lyophilized to afford **13** (33.0 mg, 92%) as a white solid: *R_f_* = 0.40 (10:1 CH_2_Cl_2_–CH_3_OH); [α]_D_^25^ –69.4 (*c* = 0.66, CH_3_OH); ^1^H NMR (600 MHz, D_2_O) δ 4.99 (d, *J* = 2.2 Hz, 1H, H-1), 4.08 (dd, *J* = 6.7, 4.1 Hz, 1H, H-3), 4.05 (dd, *J* = 4.2, 2.3 Hz, 1H, H-2), 3.95 (dd, *J* = 6.7, 3.8 Hz, 1H, H-4), 3.84 (app dt, *J* = 8.0, 4.2 Hz, 1H, H-5), 3.74 (app dt, *J* = 9.8, 6.7 Hz, 1H, octyl OCH_2_), 3.71 (dd, *J* = 11.7, 4.4 Hz, 1H, H-6a), 3.66 (dd, *J* = 11.7, 7.6 Hz, 1H, H-6b), 3.56 (app dt, *J* = 9.9, 6.6 Hz, 1H, octyl OCH_2_), 3.32 (app t, *J* = 6.9 Hz, 2H, octyl CH_2_N_3_), 1.66–1.57 (m, 4H, OCH_2_C*H*_2,_ C*H*_2_CH_2_NH), 1.43–1.32 (m, 8H, 4 x CH_2_); ^13^C NMR (151 MHz, D_2_O) δ 107.1 (C-1), 82.4 (C-4), 81.0 (C-2), 76.5 (C-3), 70.6 (C-5), 68.4 (octyl O*C*H_2_), 62.9 (C-6), 51.2 (octyl CH_2_N_3_), 28.7 (CH_2_), 28.4 (CH_2_), 28.3 (CH_2_), 28.1 (CH_2_), 26.0 (CH_2_), 25.2 (CH_2_); HRMS (ESI–TOF) *m/z* [M + Na]^+^ calcd for C_14_H_27_N_3_NaO_6_^+^ 356.1792, found 356.1796.

**^1^H NMR (600 MHz, D_2_O) and ^13^C NMR (151 MHz, D_2_O) spectra of novel compounds are shown below:**

^1^H NMR (600 MHz, D_2_O) spectrum of compound **1**


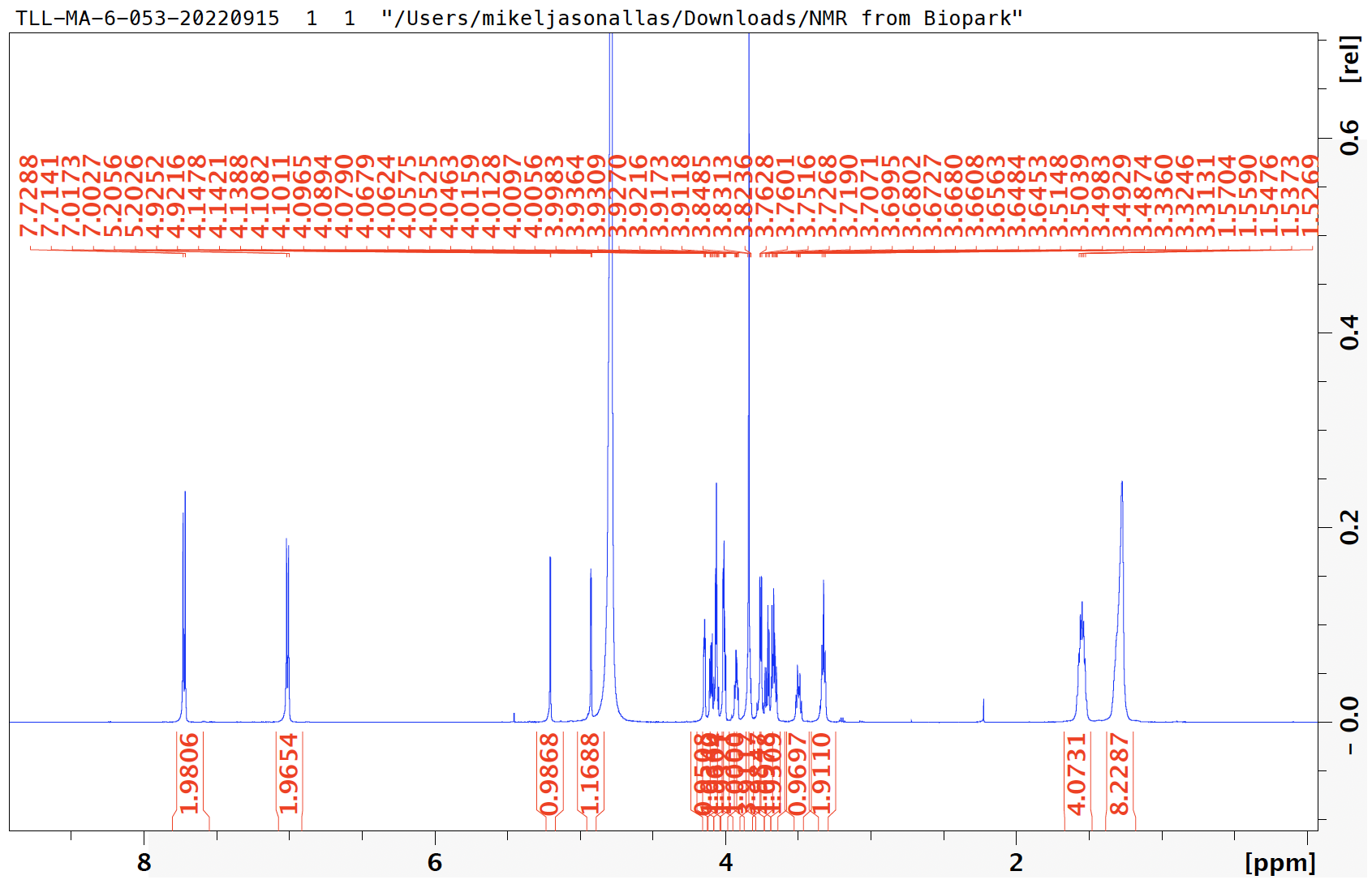


^13^C NMR (151 MHz, D_2_O) spectrum of compound **1**


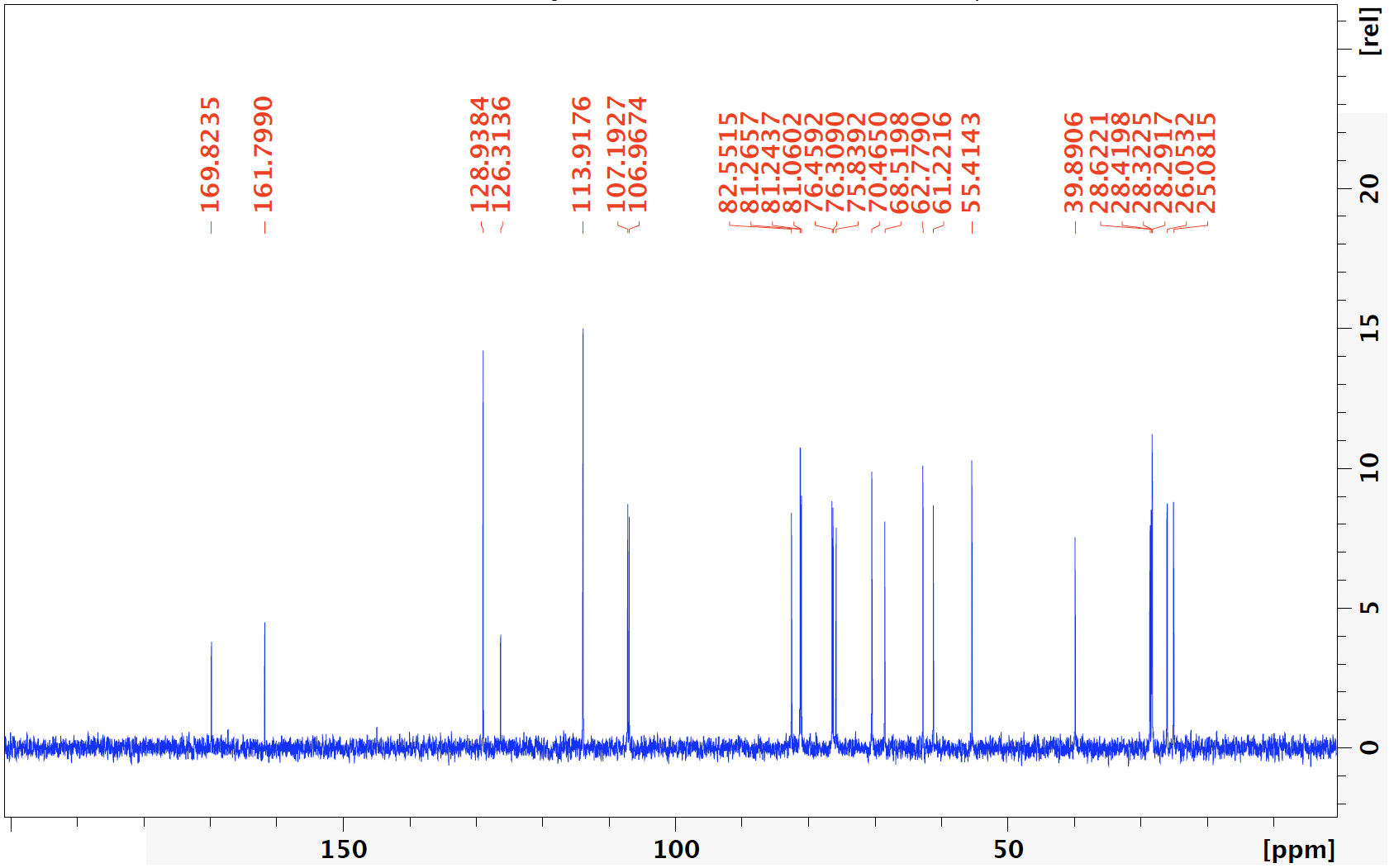


^1^H NMR (600 MHz, CD_3_OD) spectrum of compound **4**


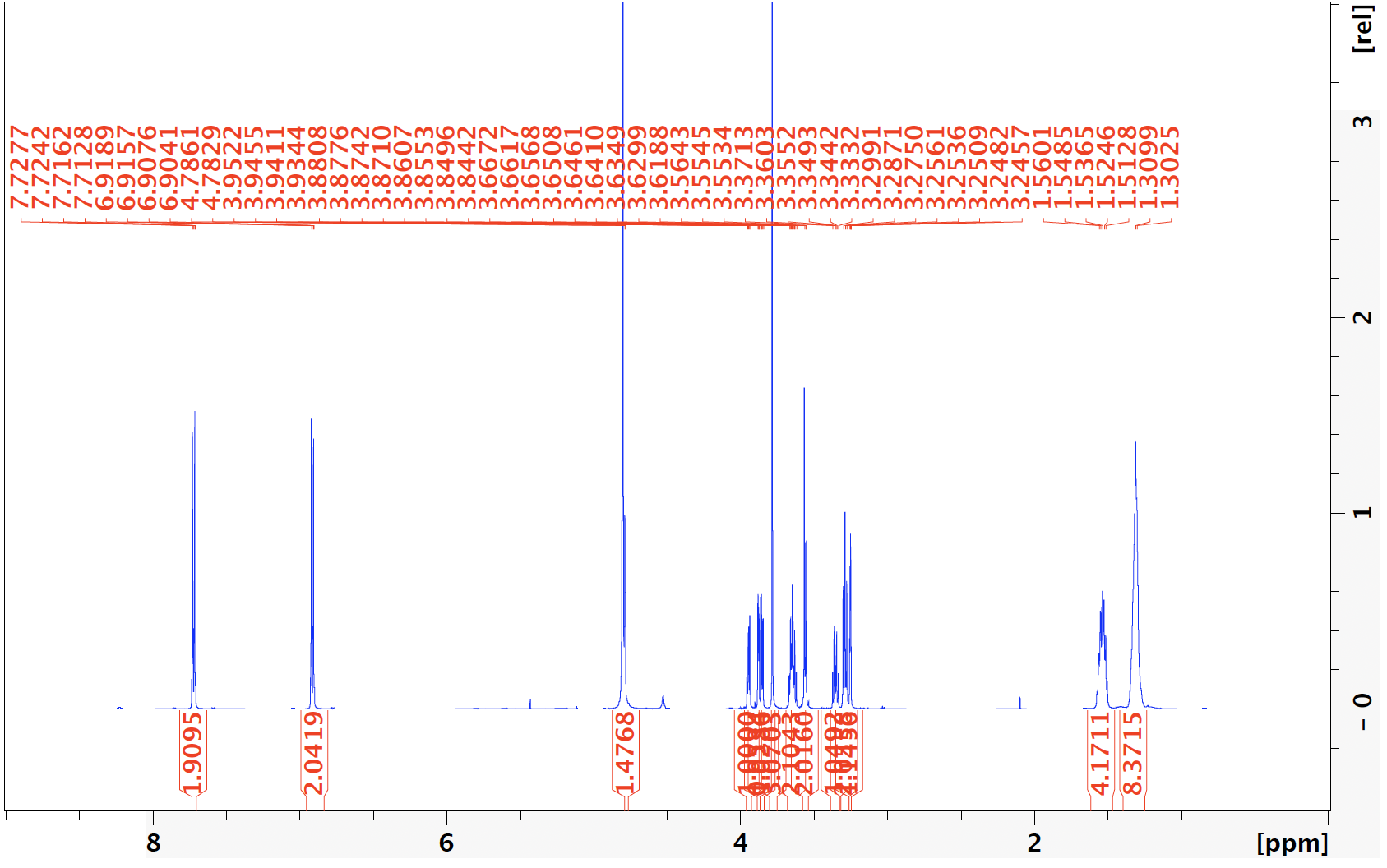


^13^C NMR (151 MHz, CD_3_OD) spectrum of compound **4**


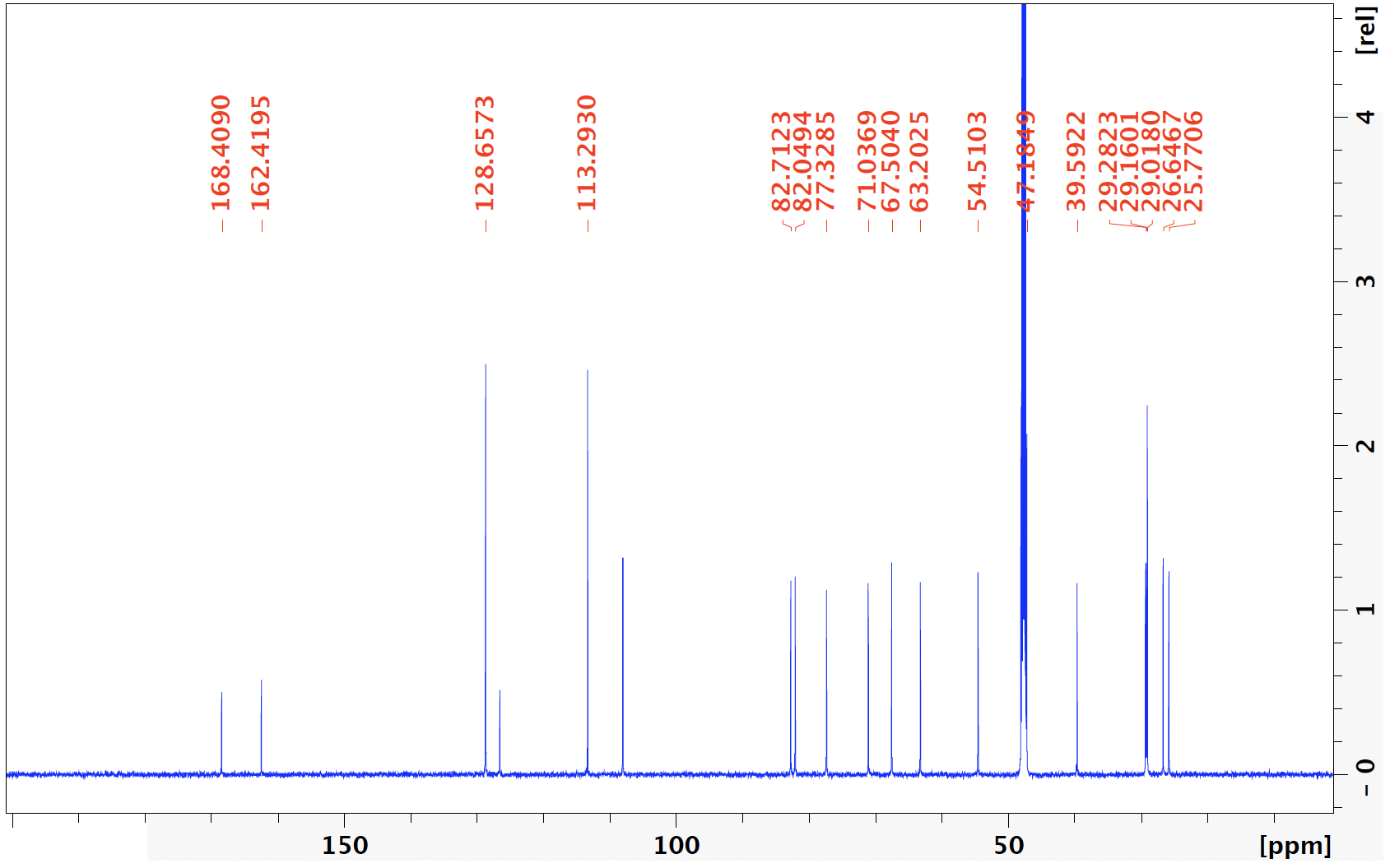


^1^H NMR (600 MHz, CDCl_3_) spectrum of compound **9**

^^
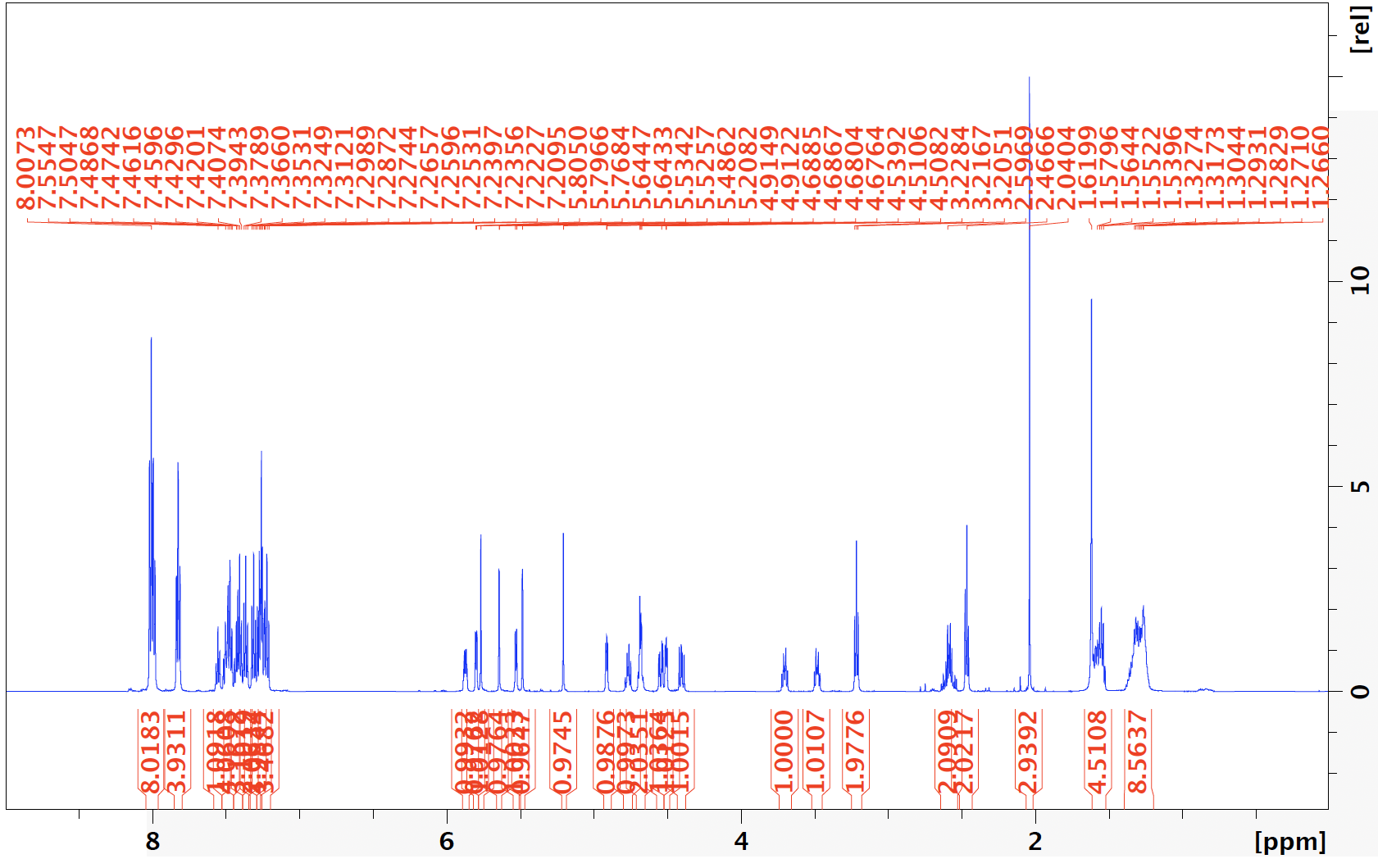


^13^C NMR (151 MHz, CDCl_3_) spectrum of compound **9**

^^
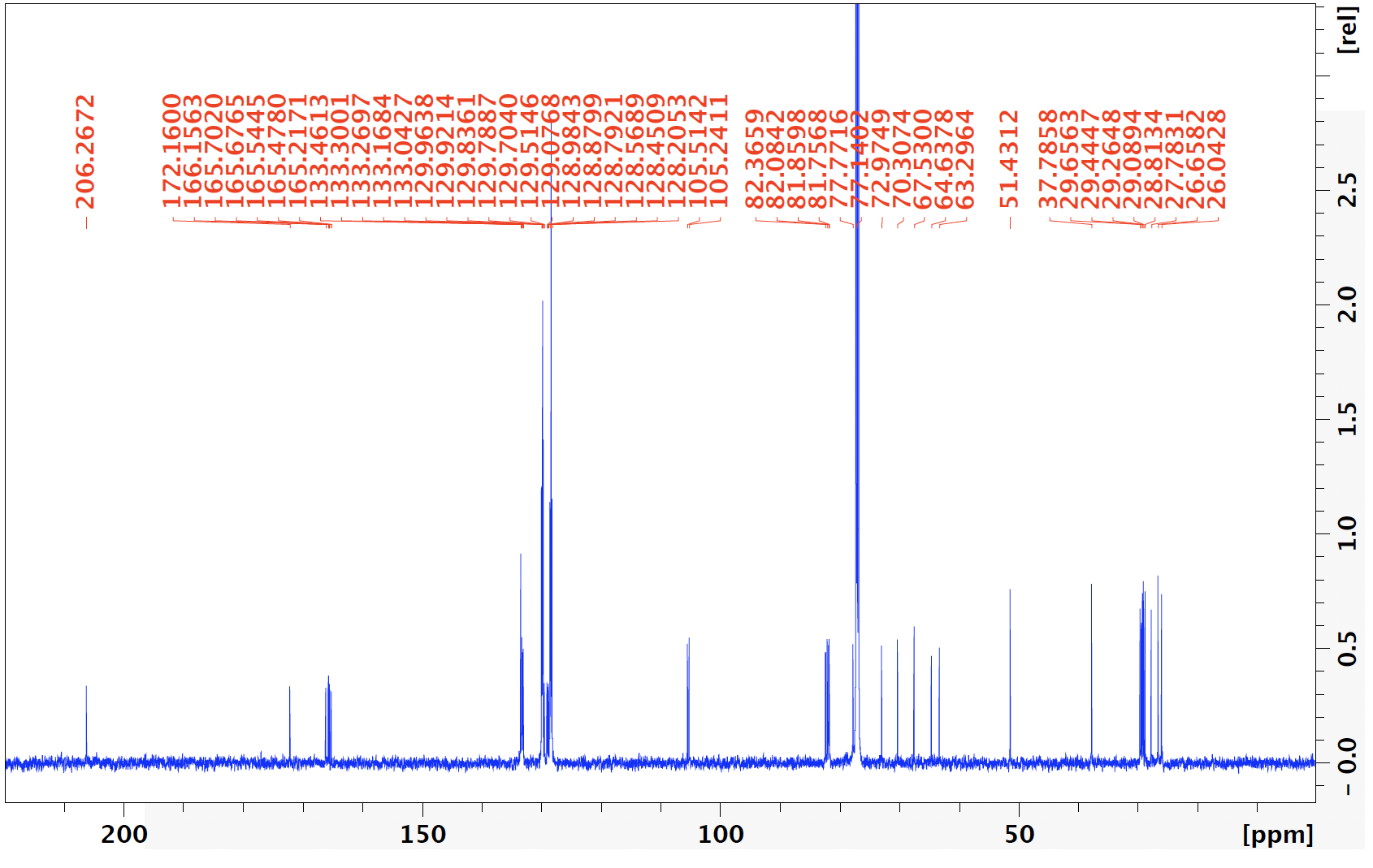


^1^H NMR (600 MHz, D_2_O) spectrum of compound **10**

^^
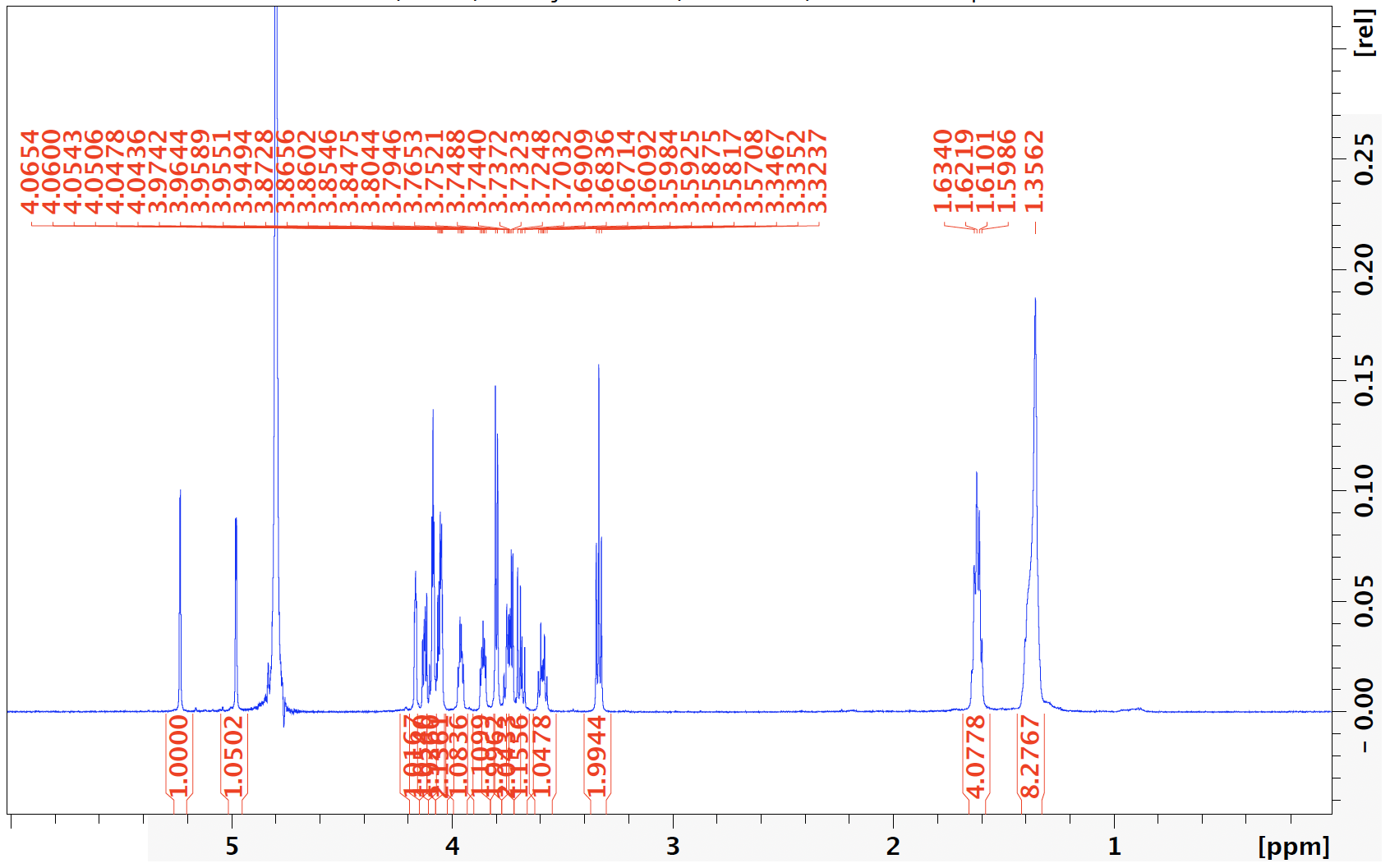


^13^C NMR (151 MHz, D_2_O) spectrum of compound **10**

^^**
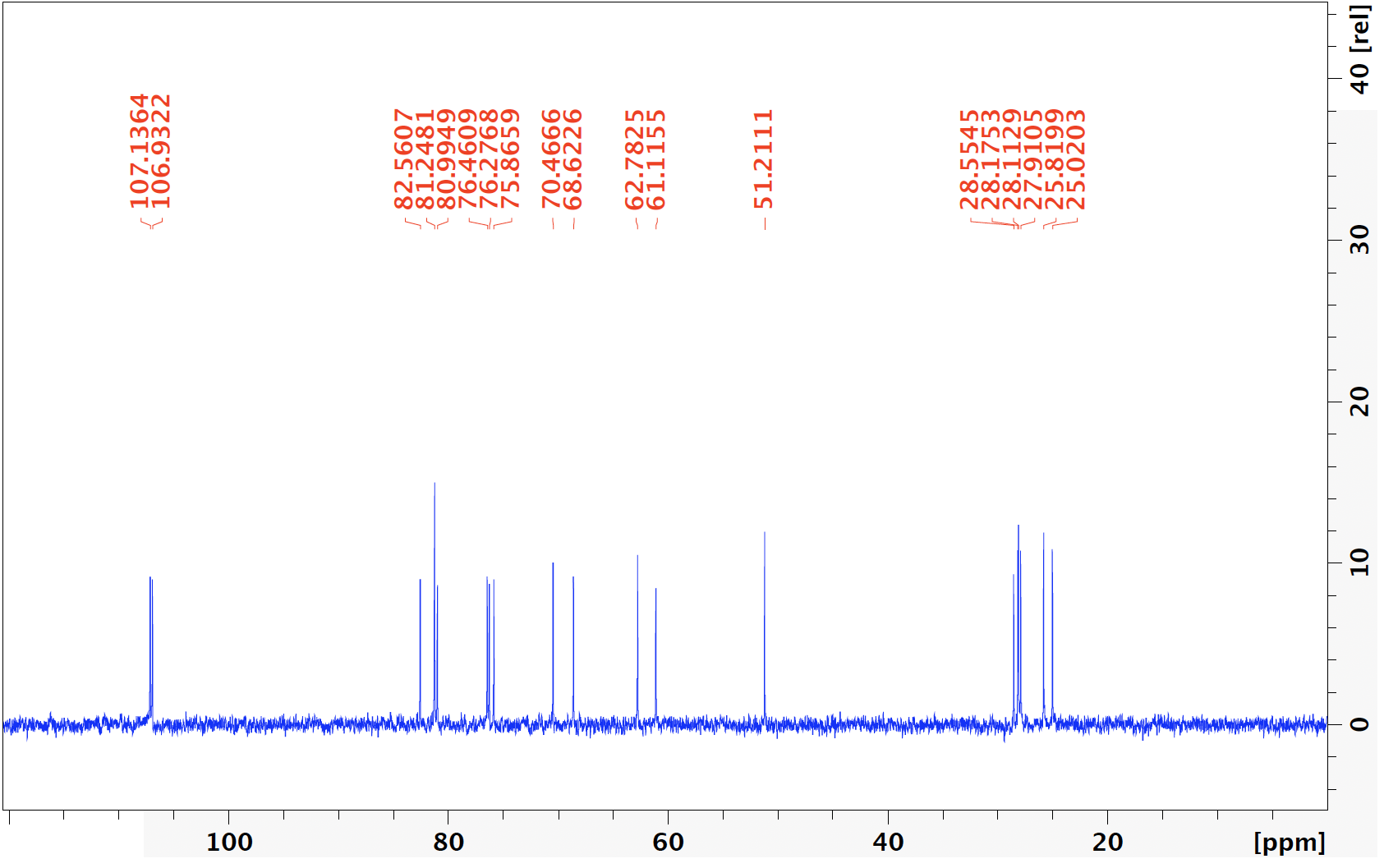
**

^1^H NMR (600 MHz, CDCl_3_) spectrum of compound **12**

^^
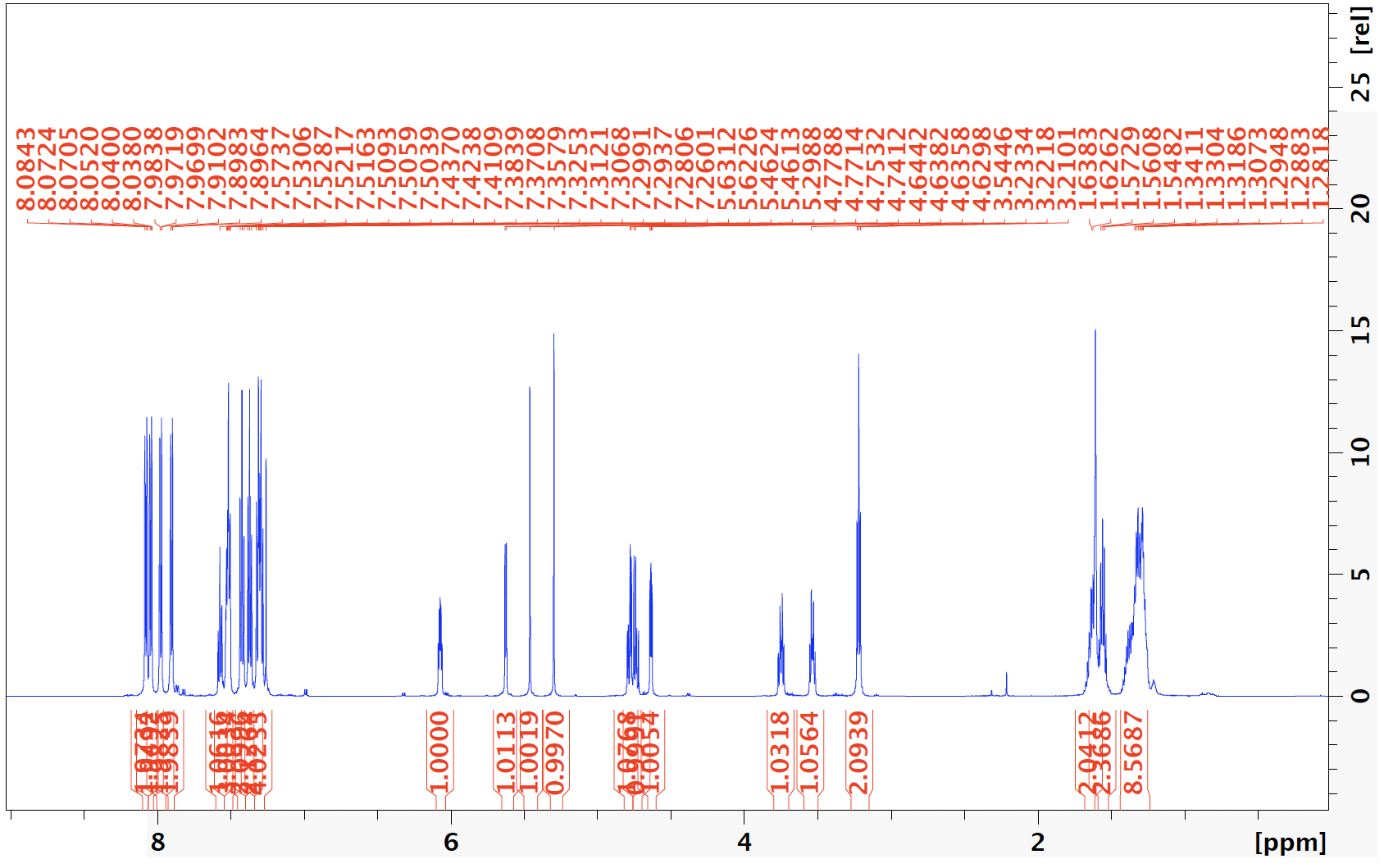


^13^C NMR (151 MHz, CDCl_3_) spectrum of compound **12**

^^**
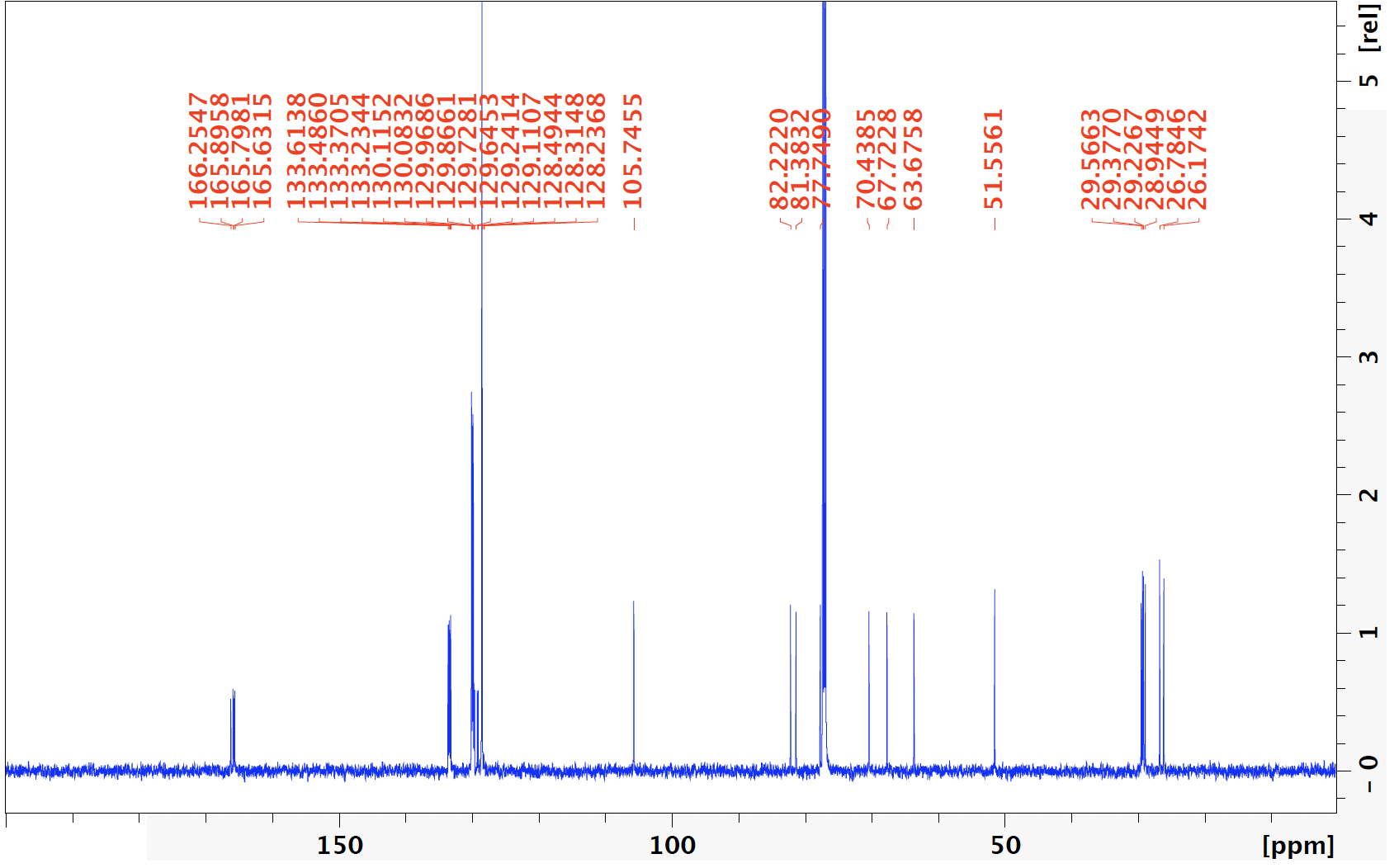
**

^1^H NMR (600 MHz, D_2_O) spectrum of compound **13**

^^
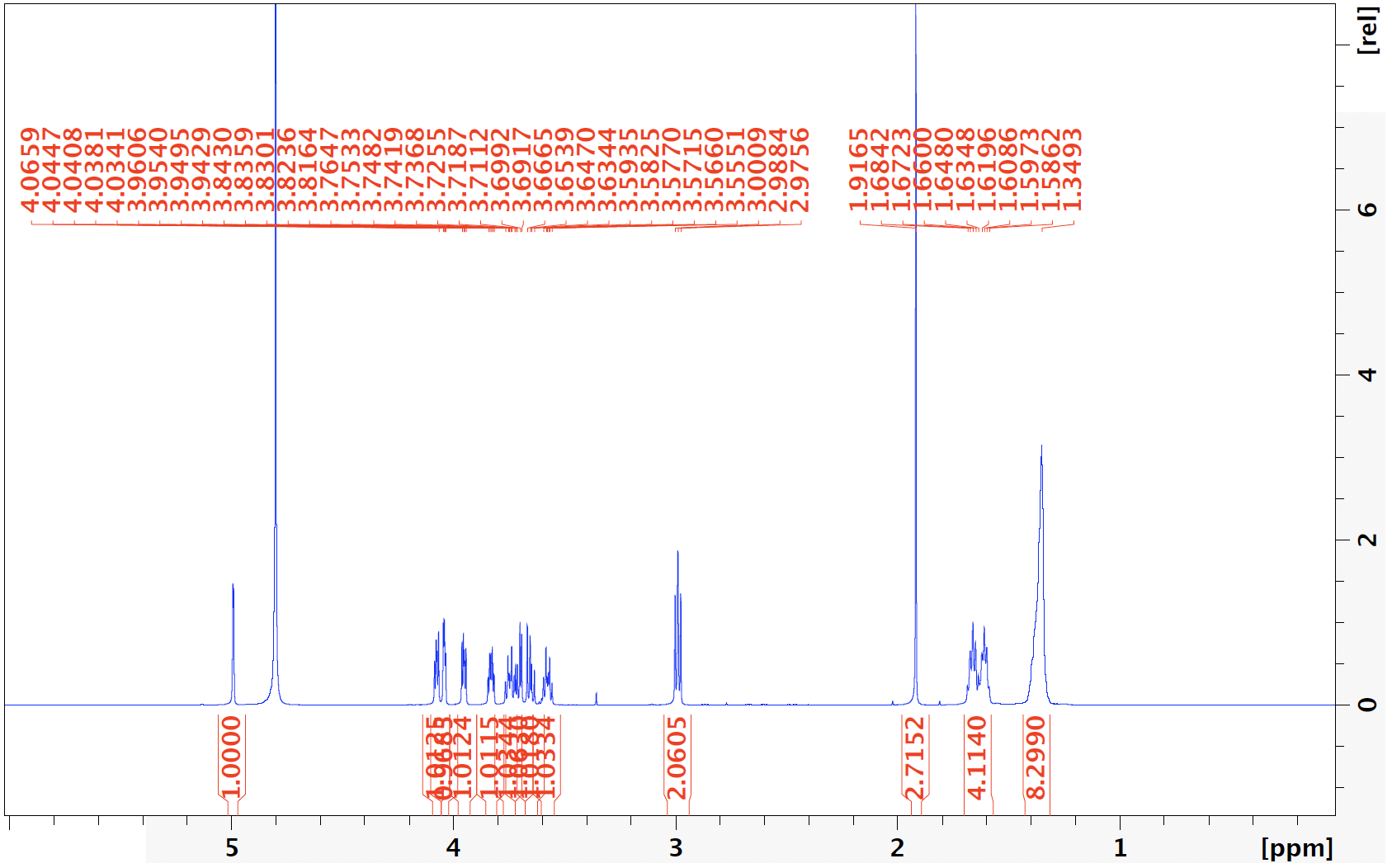


^13^C NMR (151 MHz, D_2_O) spectrum of compound **13**

^^**
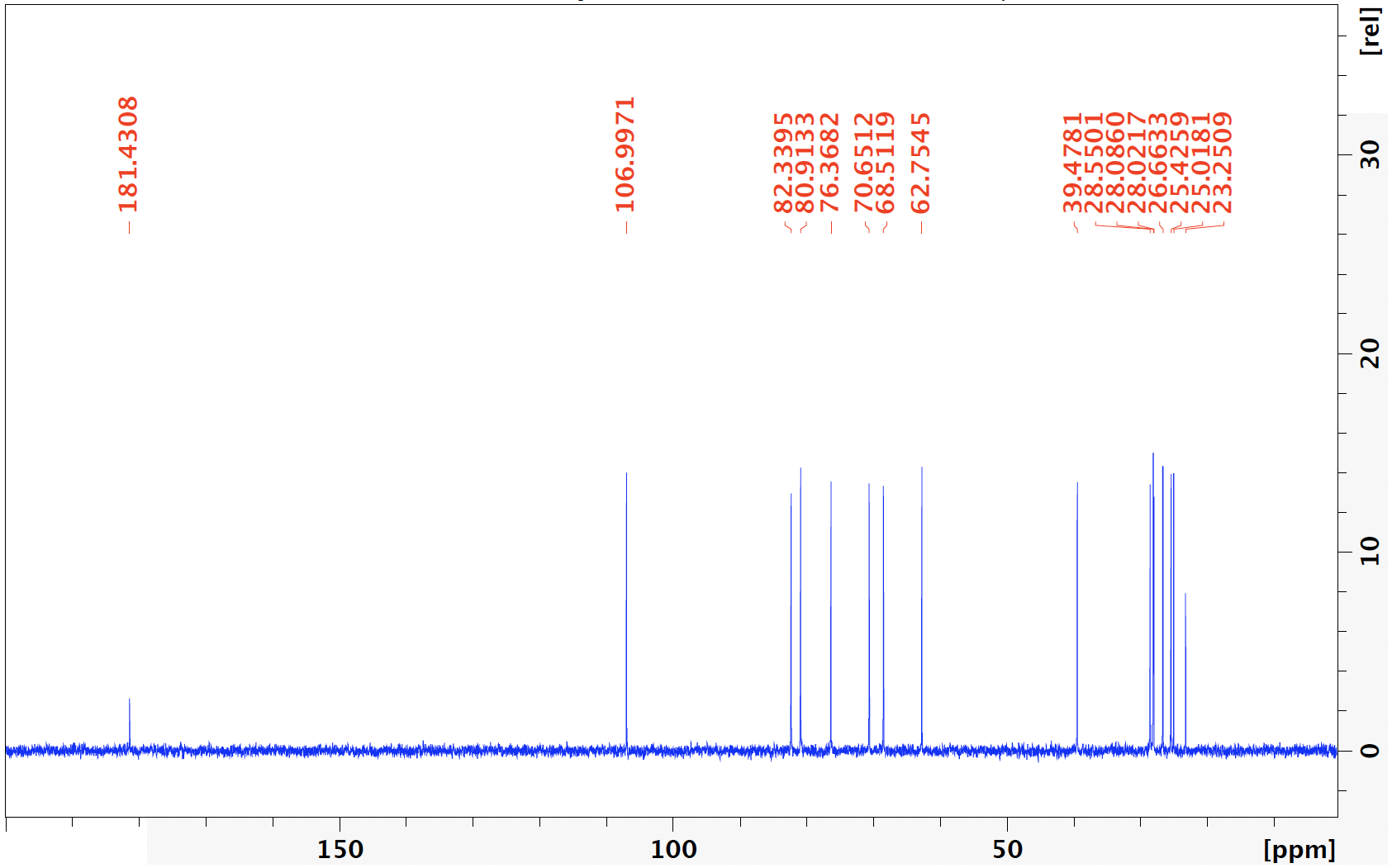
**

**SUPPORTING REFERENCES**

1. Sanders, D. A., Staines, A. G., McMahon, S. A., McNeil, M. R., Whitfield, C., and Naismith, J. H. (2001) UDP-galactopyranose mutase has a novel structure and mechanism. *Nat Struct Biol* **8**, 858-863 10.1038/nsb1001-858

2. Clarke, B. R., Ovchinnikova, O. G., Sweeney, R. P., Kamski-Hennekam, E. R., Gitalis, R., Mallette, E. *et al.* (2020) A bifunctional O-antigen polymerase structure reveals a new glycosyltransferase family. *Nat Chem Biol* **16**, 450-457 10.1038/s41589-020-0494-0

3. Wheatley, R. W., Zheng, R. B., Richards, M. R., Lowary, T. L., and Ng, K. K. (2012) Tetrameric structure of the GlfT2 galactofuranosyltransferase reveals a scaffold for the assembly of mycobacterial Arabinogalactan. *J Biol Chem* **287**, 28132-28143 10.1074/jbc.M112.347484

4. Poulin, M. B., and Lowary, T. L. (2010) Methods to study the biosynthesis of bacterial furanosides. *Methods Enzymol* **478**, 389-411 10.1016/S0076-6879(10)78019-8

5. Nassau, P. M., Martin, S. L., Brown, R. E., Weston, A., Monsey, D., McNeil, M. R. *et al.* (1996) Galactofuranose biosynthesis in *Escherichia coli* K-12: identification and cloning of UDP-galactopyranose mutase. *J Bacteriol* **178**, 1047-1052 10.1128/jb.178.4.1047-1052.1996

6. Starr, K. F., Porsch, E. A., Heiss, C., Black, I., Azadi, P., and St Geme, J. W., 3rd (2013) Characterization of the *Kingella kingae* polysaccharide capsule and exopolysaccharide. *PLoS One* **8**, e75409 10.1371/journal.pone.0075409

7. Bendaoud, M., Vinogradov, E., Balashova, N. V., Kadouri, D. E., Kachlany, S. C., and Kaplan, J. B. (2011) Broad-spectrum biofilm inhibition by *Kingella kingae* exopolysaccharide. *J Bacteriol* **193**, 3879-3886 10.1128/JB.00311-11

8. Kehl-Fie, T. E., and St Geme, J. W., 3rd (2007) Identification and characterization of an RTX toxin in the emerging pathogen *Kingella kingae*. *J Bacteriol* **189**, 430-436 10.1128/JB.01319-06

9. Montoya, N. R., Porsch, E. A., Munoz, V. L., Muszynski, A., Vlach, J., Hahn, D. K. *et al.* (2022) Surface Anchoring of the *Kingella kingae* Galactan Is Dependent on the Lipopolysaccharide O-Antigen. *mBio* **13**, e0229522 10.1128/mbio.02295-22

10. Starr, K. F., Porsch, E. A., Seed, P. C., Heiss, C., Naran, R., Forsberg, L. S. *et al.* (2016) *Kingella kingae* Expresses Four Structurally Distinct Polysaccharide Capsules That Differ in Their Correlation with Invasive Disease. *PLoS Pathog* **12**, e1005944 10.1371/journal.ppat.1005944

11. Porsch, E. A., Kehl-Fie, T. E., and St Geme, J. W., 3rd (2012) Modulation of *Kingella kingae* adherence to human epithelial cells by type IV Pili, capsule, and a novel trimeric autotransporter. *MBio* **3**, 10.1128/mBio.00372-12

12. Amann, E., Ochs, B., and Abel, K. J. (1988) Tightly regulated tac promoter vectors useful for the expression of unfused and fused proteins in Escherichia coli. *Gene* **69**, 301-315 10.1016/0378-1119(88)90440-4

13. Kelly, S. D., Allas, M. J., Goodridge, L. D., Lowary, T. L., and Whitfield, C. (2024) Structure, biosynthesis and regulation of the T1 antigen, a phase-variable surface polysaccharide conserved in many Salmonella serovars. *Nat Commun* **15**, 6504 10.1038/s41467-024-50957-y

14. Completo, G. C., and Lowary, T. L. (2008) Synthesis of galactofuranose-containing acceptor substrates for mycobacterial galactofuranosyltransferases. *J Org Chem* **73**, 4513-4525 10.1021/jo800457j

15. Li, X.-H., He, P., Liu, X.-Y., Chao, R.-B., and Wang, F.-P. (2015) Synthesis and cardiac activity evaluation of the proposed structures of fuzinoside. *Tetrahedron* **71**, 8661-8668 10.1016/j.tet.2015.09.009
